# A High-Throughput Method for Identifying Novel Genes That Influence Metabolic Pathways Reveals New Iron and Heme Regulation in Pseudomonas aeruginosa

**DOI:** 10.1128/mSystems.00933-20

**Published:** 2021-02-02

**Authors:** David G. Glanville, Caroline Mullineaux-Sanders, Christopher J. Corcoran, Brian T. Burger, Saheed Imam, Timothy J. Donohue, Andrew T. Ulijasz

**Affiliations:** a Department of Microbiology and Immunology, Loyola University Chicago, Maywood, Illinois, USA; b MRC Centre for Molecular Bacteriology and Infection, Department of Medicine, Imperial College, London, United Kingdom; c Great Lakes Bioenergy Research Center, Wisconsin Energy Institute, University of Wisconsin, Madison, Wisconsin, USA; d Department of Bacteriology, University of Wisconsin, Madison, Wisconsin, USA; University of California, Berkeley

**Keywords:** *Pseudomonas aeruginosa*, Tn-seq, biosensor, heme, heme biosynthesis, heme transport, infection, iron, metabolism, transposon sequencing, FlowSeq, Met-Seq

## Abstract

Heme is an essential metabolite for most life on earth. Bacterial pathogens almost universally require iron to infect a host, often acquiring this nutrient in the form of heme. The Gram-negative pathogen Pseudomonas aeruginosa is no exception, where heme acquisition and metabolism are known to be crucial for both chronic and acute infections. To unveil unknown genes and pathways that could play a role with heme metabolic flux in this pathogen, we devised an omic-based approach we dubbed “Met-Seq,” for metabolite-coupled transposon sequencing. Met-Seq couples a biosensor with fluorescence-activated cell sorting (FACS) and massively parallel sequencing, allowing for direct identification of genes associated with metabolic changes. In this work, we first construct and validate a heme biosensor for use with P. aeruginosa and exploit Met-Seq to identify 188 genes that potentially influence intracellular heme levels. Identified genes largely consisted of metabolic pathways not previously associated with heme, including many secreted virulence effectors, as well as 11 predicted small RNAs (sRNAs) and riboswitches whose functions are not currently understood. We verify that five Met-Seq hits affect intracellular heme levels; a predicted extracytoplasmic function (ECF) factor, a phospholipid acquisition system, heme biosynthesis regulator Dnr, and two predicted antibiotic monooxygenase (ABM) domains of unknown function (PA0709 and PA3390). Finally, we demonstrate that PA0709 and PA3390 are novel heme-binding proteins. Our data suggest that Met-Seq could be extrapolated to other biological systems and metabolites for which there is an available biosensor, and provides a new template for further exploration of iron/heme regulation and metabolism in P. aeruginosa and other pathogens.

**IMPORTANCE** The ability to simultaneously and more directly correlate genes with metabolite levels on a global level would provide novel information for many biological platforms yet has thus far been challenging. Here, we describe a method to help address this problem, which we dub “Met-Seq” (metabolite-coupled Tn sequencing). Met-Seq uses the powerful combination of fluorescent biosensors, fluorescence-activated cell sorting (FACS), and next-generation sequencing (NGS) to rapidly identify genes that influence the levels of specific intracellular metabolites. For proof of concept, we create and test a heme biosensor and then exploit Met-Seq to identify novel genes involved in the regulation of heme in the pathogen Pseudomonas aeruginosa. Met-Seq-generated data were largely comprised of genes which have not previously been reported to influence heme levels in this pathogen, two of which we verify as novel heme-binding proteins. As heme is a required metabolite for host infection in P. aeruginosa and most other pathogens, our studies provide a new list of targets for potential antimicrobial therapies and shed additional light on the balance between infection, heme uptake, and heme biosynthesis.

## Abstract (precis)

The ability to simultaneously and more directly correlate genes with metabolite levels on a global level would provide novel information for many biological platforms yet has thus far been challenging. Here, we describe a method to help address this problem, which we dub “Met-Seq” (metabolite-coupled Tn sequencing).

## INTRODUCTION

The tetrapyrrole heme is an important molecule in nature, where it serves as a cofactor in several critical enzymes, such as catalases that detoxify reactive oxygen species, and the electron transport chain, which facilitates aerobic respiration (1). In addition, heme serves as a major source of iron for most bacterial pathogens (2). Owing to its importance, most bacteria are able to synthesize heme *de novo* (1) or have dedicated systems for internalizing it from the environment (e.g., a host) (3). In the former case, there are two main routes to synthesizing heme: the C_4_ and C_5_ pathways. In the C_4_ pathway, glycine and coenzyme A (CoA), the latter a product of the tricarboxylic acid (TCA) cycle, are condensed to 5-aminolevulinic acid (ALA), the first committed heme precursor. The C_5_ pathway relies on glutamate and a two-reaction step to synthesize ALA (1), and is the only pathway harbored by most nonphotosynthetic prokaryotes (1).

Contrary to the well-conserved heme biosynthesis pathways in bacteria (and all life), heme uptake systems can differ between bacterial species yet serve the same function: to internalize environmental heme as a major source of iron. Heme uptake has been well studied in the Gram-negative pathogen Pseudomonas aeruginosa, a major cause of death in cystic fibrosis patients and source of infection in burn victims (4). In both circumstances, heme is required to cause robust disease states (5–7). P. aeruginosa possesses three known heme uptake systems, *Pseudomonas* heme uptake (Phu), heme assimilation system (Has), and hemopexin uptake (Hxu), whose outer membrane receptors then use the PhuUV inner membrane transporter and ultimately the periplasmic chaperone PhuS to traffic heme to the cytoplasm. Once internalized, PhuS then chaperones heme to a heme oxygenase, HemO (8, 9), a major regulator of heme uptake in this pathogen. HemO cleaves heme to yield biliverdin (BV) IXδ, enabling iron extraction (10). P. aeruginosa also possesses another heme oxygenase, BphO, that serves to convert heme to BV IXα, which is then attached to the phytochrome light receptor to enable far-red-light detection, and the subsequent modulation of biofilm formation and possibly virulence (11).

To date, most heme-related studies in P. aeruginosa have focused on the aforementioned heme uptake and iron acquisition pathways (for reviews see references 4, 5, 8, and 12). In comparison, the influence of heme biosynthesis on heme uptake and the general maintenance of intracellular heme levels has been neglected (2). One reason for this could be that heme biosynthesis and its genes are essential and, therefore, difficult to study. In an attempt to address this issue and discover novel genes and metabolic pathways involved in the maintenance of total intracellular heme levels, we devised a heme biosensor based on a phytochrome light receptor protein architecture (13–17) as per Nobles et al. (18). After testing the efficacy of our biosensor, we then built on existing Tn-coupled “FlowSeq”-based studies (e.g., fluorescence-activated sorting of transposon mutants coupled with insertion site sequencing [FAST-INSeq] [19], transposon-directed insertion sequencing enrichment [TraDISort] [20], fluorescence-activated cell sorting with NGS for persister physiology [Persister-FACSeq] [21], and others [22, 23]) to enable the identification of genes *en masse* that affect the levels of a desired metabolite, which we dub here metabolite-coupled Tn-sequencing (Met-Seq).

After three rounds of fluorescence-activated cell sorting (FACS) enrichment, Met-Seq identified 188 genes that significantly diminished the biosensor signal. Results included several known iron/heme regulatory genes such as *dnr*, which positively controls heme biosynthesis by regulation of both *hemA* and *hemF* transcription in P. aeruginosa (24, 25). However, most genes that we identified had not previously been associated with heme/tetrapyrrole regulation, including genes involved in siderophore synthesis, several predicted small RNAs (sRNAs)/riboswitches, central metabolic pathways, and virulence delivery systems and their effectors, suggesting that there is a regulatory connection between these pathways and the maintenance of intracellular heme levels. We validate that *dnr* and four novel genes identified by Met-Seq affect intracellular heme levels in P. aeruginosa, namely, genes encoding an extracytoplasmic function (ECF) sigma factor, a lipid taxis and uptake system, and two similar proteins of unknown function which both contained a predicted antibiotic monooxygenase (ABM) domain (PA0709 and PA3390). Finally, we demonstrate that the ABM domain-containing proteins PA0709 and PA3390 are both novel heme-binding proteins in this pathogen.

## RESULTS

### Construction and testing of a heme biosensor in P. aeruginosa.

**FIG 1:**
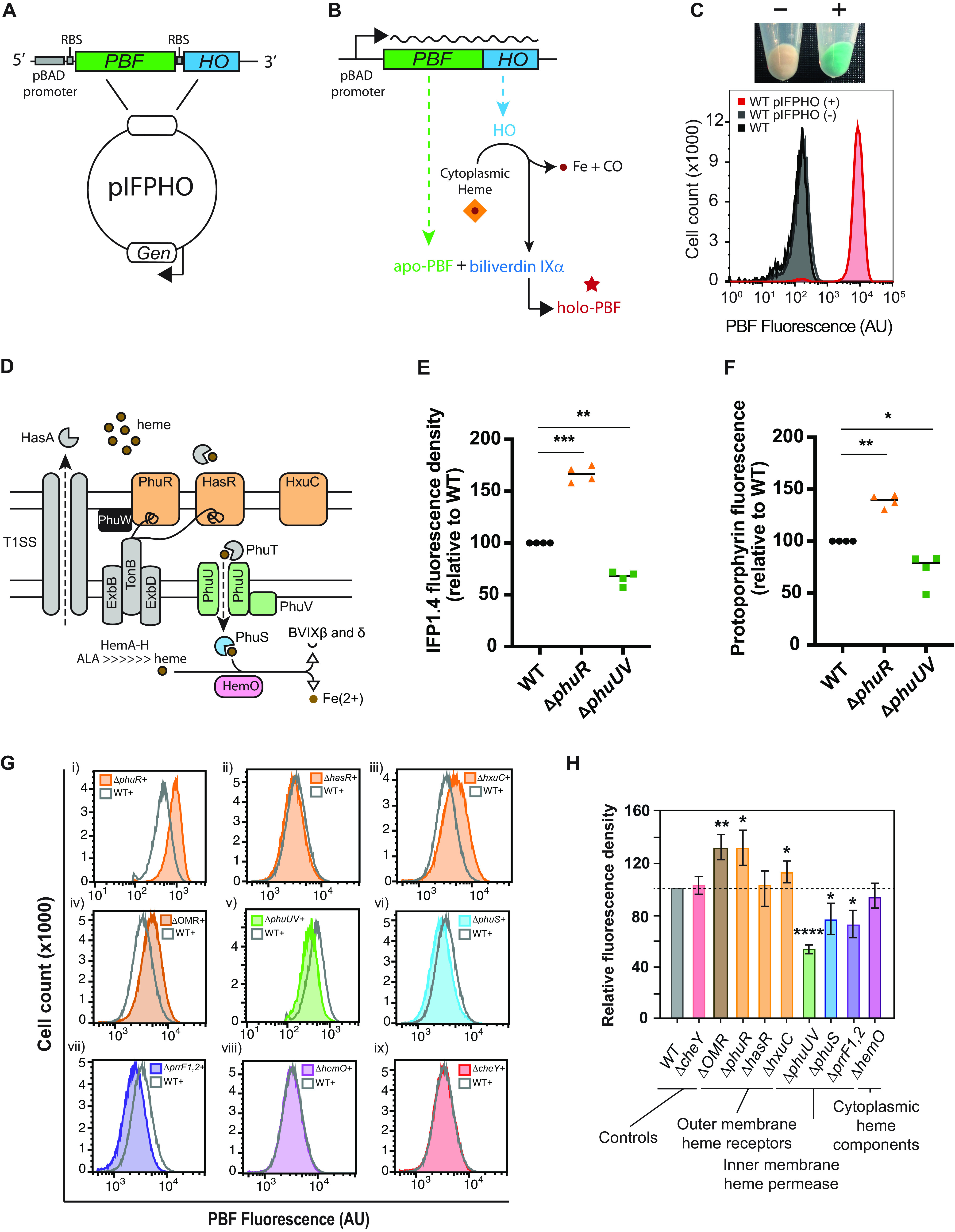
PBF-HO heme biosensor. Map of the pIFPHO plasmid, constructed from parent plasmid pSB109 (A) and schematic diagram depicting biosensor function (B). (C) (Top) Cell pellets of MPAO1 pIFPHO grown in M9 minimal medium plus 5 μM heme without (−) and with (+) 0.2% arabinose. (Bottom) Flow cytometric analysis of MPAO1 WT and MPAO1 pIFPHO without (−) or with (+) 0.2% arabinose. Data are representative of two biological repeats. (D) Schematic representation of components of the known heme uptake and processing systems of P. aeruginosa. Biosensor (IFP1.4) fluorescence density (E) and protoporphyrin fluorescence (F) as measured according to reference 30 after 14 h of growth in M9 plus 5 μM heme and 0.2% arabinose. Both fluorescence density and protoporphyrin fluorescence data were derived from the same samples for optimal comparison. Individual data points are plotted. The horizontal lines indicate the median values from four biological repeats. Flow cytometry analysis of PBF-expressing MPAO1 mutants (G) and the mean (± standard deviation [SD]) relative fluorescence density (H) at the 11-h time point after growth in M9 plus 5 μM heme and 0.2% arabinose. Three biological repeats are shown as a percentage of WT fluorescence. Statistically significant differences in panels E and F were determined using a one-sample *t* test with Wilcoxon test; statistically significant differences in panel H were determined using a one-sample *t* test. *, *P* < 0.05; **, *P* < 0.01; ***, *P* < 0.001; ****, *P* < 0.0001.

To construct our phytochrome heme biosensor, we used an arabinose-inducible replicating parent expression vector (pSB109) to express the phytochrome-based fluorophore (PBF) protein (IFP1.4 [16]) and a bacterial heme oxygenase (HO) as a synthetic operon, resulting in vector pIFPHO (Fig. 1A). We reasoned that when induced with arabinose, free heme would be degraded to BV IXα, which would, in turn, be incorporated into the PBF to give near-infrared (NIR) fluorescence (Fig. 1B) (13, 15, 16, 26). To test this hypothesis, we transformed wild-type (WT) MPAO1 cells with either pIFPHO, pIFPHO lacking the heterologous HO (pIFP), or the empty parent vector (pSB109) and then grew cells in the presence or absence of arabinose and measured NIR fluorescence. Only cells expressing the heterologous HO fluoresced and resulted in the expected dramatic change in cellular absorbance (Fig. 1C; see also Fig. S1A and B in the supplemental material) (27, 28), indicating that expression of a heterologous HO is required for biosensor detection and that virtually all of the cellular BV IXα was being provided by the pIFPHO heme biosensor-expressing plasmid. In addition, the fluorescence signal was stable over time (Fig. S1C and D). Importantly, WT MPAO1 grew comparably with cells harboring either empty plasmid (pSB109) or the biosensor plasmid (pIFPHO) in minimal medium supplemented with 5 μM heme and induced with arabinose (Fig. S1E). Since these conditions were identical to conditions used in our Met-Seq screen (see below), these data suggest that the biosensor does not sufficiently alter the bacterial metabolism so as to produce a stress response, and that excess carbon monoxide, an inhibitor of respiration (29), was not produced by the HO at sufficient concentrations to affect growth.

10.1128/mSystems.00933-20.1 FIG S1 Fluorescence expression and stability of the biosensor plasmid. Fluorescence densities of MPAO1 containing various PBF reporter constructs after 15 h of growth in M9 plus 5 μM heme (A) and 7 h growth in LB medium (B). Fluorescence curves of MPAO1 containing various PBF reporter constructs grown M9 plus 5 μM heme (C) or LB medium (D). Curves are a single experiment representative of at least three biological repeats. (E) Growth of MPAO1 WT harboring either no plasmid, the empty pSB109 parent plasmid, or pIFPHO. Means ± standard errors of the means (SEMs) from three biological repeats are shown. ****, *P* < 0.0001 versus WT by one-way analysis of variance (ANOVA) with Tukey’s multiple-comparison posttest; AU, arbitrary units; *A*_600_, absorbance at 600 nm. Relative heme concentrations of MPAO1 (WT), Δ*phuR*, and Δ*phuUV* mutants as measured by protoporphyrin fluorescence when harboring pIFPHO without the addition of arabinose (F) or in the absence of the biosensor (pSB109 parent plasmid only) (G). Horizontal lines represent the medians from 4 biological repeats. Individual data points are plotted. *, *P* < 0.05; ns, not significant compared to WT as determined by a one-sample *t* test with Wilcoxon test. (H) Western blot of FLAG-HO showing the induction of the HO with and without arabinose addition. WT, Δ*phuR*, and Δ*phuUV* strains harboring pIFPHO were grown in M9 plus 5 μM heme, and cells were harvested after 14 h of growth. Download FIG S1, EPS file, 2.0 MB. Copyright © 2021 Glanville et al. 2021 Glanville et al. This content is distributed under the terms of the Creative Commons Attribution 4.0 International license.

We then examined if the biosensor fluorescence paralleled actual heme levels in the cell by using a standard fluorescence assay for the detection of intracellular heme (30). This was initiated by deleting the major heme uptake system (Phu [31]), outer membrane receptor PhuR, or the inner membrane transporter system (PhuUV) (Fig. 1D) and then introducing the biosensor plasmid. After culturing, both fluorescence of the biosensor and total cellular heme (through measurement of protoporphyrin fluorescence [30]) were measured for comparison (Fig. 1E and F, respectively). Results showed that the relative differences in biosensor fluorescence between the WT and deletion mutants paralleled the relative differences in intracellular heme levels, with the Δ*phuR* strain showing a marked increase in protoporphyrin-derived fluorescence compared to that for the WT, and the Δ*phuUV* strain showing a marked decrease (Fig. 1E and F). These trends also held true when cells were grown under the same conditions but without the addition of arabinose (Fig. S1F). However, when grown in the absence of the biosensor (pSB109), the Δ*phuUV* mutant displayed a statistically significant increase in relative heme levels instead of the decrease observed when the cells harbored the pIFPHO plasmid, regardless of whether arabinose was added (Fig. S1G). This difference could be explained by the leaky expression of the HO from the pIFPHO plasmid, as determined by anti-FLAG Western blotting (Fig. S1H). Taken together, these data suggest that expression of the IFPHO biosensor is an accurate read-out of intracellular free (available) heme. However, expression of the biosensor clearly altered heme homeostasis, which could then become amplified through deletion of certain genes (e.g., *phuUV*).

We then sought to test the response of the reporter strain to heme uptake or biosynthesis by the extracellular addition of either heme or the first committed precursor to heme biosynthesis, 5-aminolevulinic acid (ALA) (1), respectively. As expected, reporter cells grown in LB supplemented with increasing concentrations of ALA showed a concomitant increased PBF fluorescence density in a dose-dependent manner (see Fig. S2A to C), indicating that the biosensor detected heme biosynthesis increases. On the other hand, reporter cells cultured in medium containing heme as the sole iron source resulted in a clear dose-dependent reduction in biosensor fluorescence density (Fig. S2D to F), suggesting that the presence of extracellular heme results in decreased available free intracellular heme. These results suggest that the addition of extracellular heme might be suppressing the biosynthesis of ALA/heme, a phenomenon that was previously documented in Escherichia coli (32). Collectively, our data suggest that it is biosynthesized heme which is primarily being degraded by the HO heterologously provided by biosensor plasmid and therefore “seen” by our PBF reporter.

10.1128/mSystems.00933-20.2 FIG S2 Reporter fluorescence is responsive to addition of heme and ALA. Representative growth curves (A) and fluorescence density (AU/*A*_600_) (B) versus time (h) curves of MPAO1 pIFPHO grown in LB containing 0 to 500 μg/ml ALA. The inset shows the average fluorescence density of cells normalized to 0 μg/ml ALA after 15 h. The means from four biological repeats ± SDs are shown. (C) Average fluorescence densities plotted against log_10_ [ALA (μg/ml)]. Means from four biological repeats ± SDs are shown. Representative growth curves (D) and fluorescence density curves (E) of MPAO1 pIFPHO grown in M9 minimal medium containing 0.01 to 10 μM hemin. The inset shows the average fluorescence density of cells normalized to 10 μM heme after 20 h. Means from four biological repeats ± SDs are shown. (F) Average fluorescence densities plotted against log_10_ [heme (μM)]. Means from four biological repeats ± SDs are shown. In panels B and E: *, *P* < 0.05; **, *P* < 0.01; ***, *P* < 0.001; ****, *P*< 0.0001 by a one-sample *t* test. Download FIG S2, EPS file, 1.5 MB. Copyright © 2021 Glanville et al. 2021 Glanville et al. This content is distributed under the terms of the Creative Commons Attribution 4.0 International license.

To further validate that our biosensor was responsive to changes in intracellular heme homeostasis, we deleted all three of the known outer membrane (heme) receptors (OMRs; PhuR, HasR, and HxuC [8, 33]), the PhuUV inner membrane transporter, the intracellular heme chaperone PhuS (9, 34), and the main P. aeruginosa heme oxygenase, HemO (10). We also obtained a PAO1 strain lacking the iron regulatory sRNA *prrF1/2*, which is known to influence the expression of heme-related proteins involved in both biosynthesis and uptake in P. aeruginosa (35–37) (Fig. 1D). A strain lacking the chemotaxis gene *cheY* was generated as a negative control.

Most mutants demonstrated varied fluorescence compared to that of WT cells, while deletion of *cheY* had no significant effect (Fig. 1G and H). Deletion of the OMR genes *phuR* (as previously observed) (Fig. 1E), *hxuC*, or all three OMRs (*phuR*, *hxuC*, and *hasR*; the ΔOMR strain) resulted in an elevated reporter signal. However, the signal was not affected in the Δ*hasR* mutant, a gene that has been previously shown to not significantly contribute to hemin uptake under these conditions (31). Collectively, these results may indicate a regulatory connection between OMR synthesis and heme biosynthesis, a link which was previously suggested to exist in other pathogens (2). In contrast, deletion of cytoplasmic regulator PhuS resulted in lower signal than that from the WT, similar to deletion of genes that encode the PhuUV inner membrane components. Deletion of the iron/heme regulatory sRNA *prrF1/2* also resulted in a marked decrease in signal and added further verification to previous studies which have indicated that *prrF1/2* could influence heme-related pathways (35, 37, 38). Finally, the deletion of *hemO*, which has been shown to control heme internalization (10), had no significant effect on intracellular heme concentrations as measured by our biosensor. This result presents further evidence that the biosensor primarily detects free synthesized heme rather than bound/chaperoned heme internalized by the heme uptake systems, which HemO was previously shown to control (10).

As a final test to validate and quantify the sensitivity of our assay for Met-Seq, WT MPAO1 cells expressing the biosensor were spiked with the brighter biosensor-expressing *ΔphuR* strain at different ratios. Addition of the *ΔphuR* fluorescent strain resulted in a concomitant dose-dependent increase in cells gated in a bright “*ΔphuR”* fluorescence gate (see Fig. S3A to C). These data demonstrate that small populations of cells with an altered PBF fluorescence phenotype may be identified by flow cytometry using our PBF biosensor system and could therefore be isolated by FACS.

**FIG 2:**
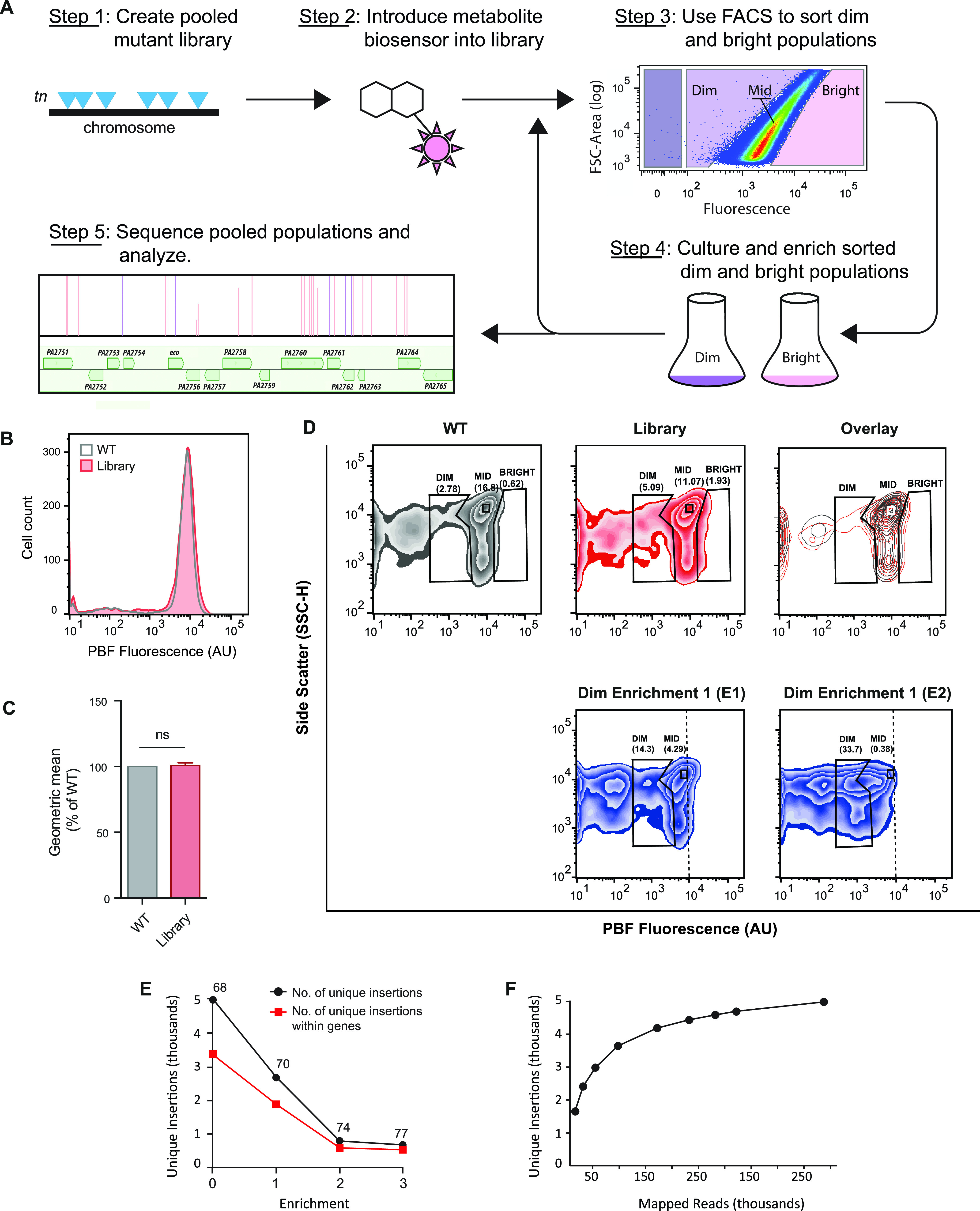
Met-Seq protocol and flow analysis. (A) Schematic diagram of the Met-Seq stepwise protocol. (B) Flow cytometric analysis of the PBF-expressing WT MPAO1 cell population (gray line) and the pooled Tn library population (red line) after 14 h of growth in M9 plus 5 μM heme and 0.2% arabinose. Histogram is representative of three biological repeats. (C) Geometric mean from three biological repeats as a percentage of WT fluorescence from the data in panel B. Means ± SDs from three biological repeats are shown. ns, not significant as determined by a one-sample *t* test. (D) Initial unsorted WT pIFPHO_AR and library pIFPHO_AR populations from panel B shown as individual zebraplots and as a contour plot of these data overlaid (overlay). The E1 and E2 dim enrichment populations are also shown as zebra plots for comparison. Numbers under the gate names indicate the percentages of population events falling into that gate. (E) Number of unique Tn insertions in the library and E1 to E3 dim populations. Enrichment 0 represents the original Tn library harboring the pIFPHO_AR biosensor. Numbers above the points indicate the percentages of total unique insertions present within both known and predicted open reading frames. (F) Unique insertions versus mapped reads in the E3 population showing that sequencing coverage was sufficient for the library complexity. A one-sample analysis in TSAS (41) was employed to identify the number of unique Tn insertions at each depth (by random sampling at each depth), which were graphed.

10.1128/mSystems.00933-20.3 FIG S3 Met-Seq controls. Flow cytometric analysis of WT pIFPHO_AR and Δ*phuR* pIFPHO_AR (Δ*phuR*) grown in M9 plus 5 μM heme for 14 h. (A) Dot plots of pure WT and Δ*phuR* culture fluorescence (top) and WT diluted 1:1 and 1:10 with Δ*phuR* culture (bottom). Populations shown are gated based on forward and side scatter. The numbers indicate the population percentages falling into the shown “*phuR*” gate. (B) Histogram of WT (grey) and Δ*phuR* (red) populations. (C) Bar chart displaying the percentages of the populations falling into the gate from panel A (i.e., the “*phuR* gate”). (D) Regrowth of E. coli harboring plasmids isolated from “dim” colonies. E. coli strains harboring pIFPHO_AR plasmids isolated from 18 E3 population randomly selected dim colonies (1 to 18) and WT P. aeruginosa MPAO1 (WT) for comparison were grown in LB. Fluorescence density was assessed after 10 h growth. Strains 12 and 17 (indicated with red arrowheads) showed a severely decreased fluorescence density, suggesting these plasmids harbor mutations rendering them nonfluorescent. Means ± SDs from three technical repeats are shown. Bars are representative of three technical repeats. Histogram (E) and Zebra plots (F) of WT “dim,” “mid,” and “bright” populations after parallel treatments as described for Fig. 2. Flow cytometric analysis showed no differences between (i) mid and dim and (ii) mid and bright collected cells. Download FIG S3, EPS file, 2.4 MB. Copyright © 2021 Glanville et al. 2021 Glanville et al. This content is distributed under the terms of the Creative Commons Attribution 4.0 International license.

### Library construction and Met-Seq.

After validation of the PBF reporter strain, we sought to discover novel genes involved in heme metabolism using the Met-Seq method (illustrated in Fig. 2A). This was accomplished by first creating a Tn mutant library using the P. aeruginosa strain MPAO1 (39) followed by introduction of a modified pIFPHO biosensor plasmid (pIFPHO_AR; see Materials and Methods for details) into the pooled library. We then compared fluorescence of the pooled Tn library population to that that of WT MPAO1 cells by using flow cytometry. As expected, the mean fluorescence values were similar (Fig. 2B and C); however, slightly greater proportions of library transformants were noted in the defined “dim” and “bright” population gates (Fig. 2D, top three panels), suggesting that the library contained a distinct set of Tn insertions resulting in differential reporter fluorescence. Cells which appeared within the dim and bright populations were then sorted and collected by FACS, propagated, and sorted once again for enrichment as per the Met-Seq protocol (see Fig. 2A).

Following the initial round of enrichment (E1), the dim cells showed a moderate enrichment (5.09% to 14.3% of the library falling into the dim gate), which then increased more substantially to 33.7% after the second (E2) enrichment (Fig. 2D). Cells falling into the dim gate in E2 were then collected by FACS in a final (third) enrichment (E3). Isolation of biosensor plasmids from randomly selected colonies in the E3 population revealed that 2 of 18 tested clones (11%) resulted in no detectable fluorescent signal when introduced into E. coli DH5α (Fig. S3D), strongly suggesting these colonies had biosensor plasmids which had incurred a mutation that rendered them “dark” rather than a bona fide Tn insertion. In contrast to the dim collected cells, the bright gate did not show an appreciable number of divergent events (1.93%) (Fig. 2D), nor did it exhibit a measurable enrichment (data not shown), and was therefore not pursued further. As a final control to ensure that the enrichment of the dim population in the pooled library was due to Tn insertions in heme-related genes and not due to random genetic variation, WT MPAO1 cells harboring the pIFPHO_AR biosensor plasmid were gated in parallel and enriched using the same criteria applied to the pooled Tn library. As expected, no enrichment occurred (Fig. S3E and F).

### Analyses of the Tn library.

To determine the genomic location of Tn insertions in cells enriched in the dim population, the original library and the three dim enrichment populations harboring pIFPHO_AR (library and E1 to -3, respectively) were analyzed using Tn sequencing (Tn-seq) (40, 41). Sequencing revealed that the pooled Tn library harbored 4,988 unique insertions, of which 3,385 were within the 1,775 annotated MPAO1 open reading frames (ORFs) (of 5,570 total ORFs). Overall, data indicated insertions in 31.8% of the total P. aeruginosa MPAO1 predicted ORFs. Approximately 12% of MPAO1 genes are essential in rich medium (LB) (39). However, because we used minimal medium in this study, this percentage could be greater. Even so, our Tn library missed 3,418 genes, or 61.4%, indicating that our coverage was not saturating. However, as many of the Tn insertions obtained were within operons and promoter (regulatory) regions, we reasoned that such insertions could disrupt the function of several genes simultaneously (i.e., operon transcript disruption), thereby enhancing our overall genome coverage. Nevertheless, several known genes involved in heme uptake and metabolism (e.g., several *has*, *phu*, and *hxuC* genes) were absent from our Tn library and therefore could not be enriched for as internal controls. A list of known heme-related genes and their Tn insertions in our library is shown in Table 1.

**TABLE 1:** Known heme uptake, regulatory, and synthesis proteins in P. aeruginosa

Gene	Locus no.^*a*^	Enzyme or function	No. of Tn inserts in original library	Reference(s)
Uptake				
*hxuA*	PA1302	Heme outer membrane receptor		148
*hxuR*	PA1301	Anti-sigma factor		70, 148
*hxuI*	PA1300	ECF sigma factor		70, 148
*hasD*	PA3406	Transport protein		149
*hasA*	PA3407	Extracellular heme binding (hemophore)		149
*hasR*	PA3408	Heme outer membrane receptor (sensing)	1	149
*hasE*	PA3405	Membrane fusion protein		149
*hasF*	PA3404	Outer membrane protein		149
*hasS*	PA3409	Anti-sigma factor		149
*hasI*	PA4310	ECF sigma factor		149
*phuR*	PA4710	Major heme outer membrane receptor	5	149
*phuU*	PA4707	Inner membrane heme transporter	1	149
*phuV*	PA4706	Inner membrane heme transporter	1	149
*phuW*	PA4705	Possible PhuR auxiliary protein		149
*phuS*	PA4709	Heme trafficking	1	149
*phuT*	PA4708	Heme trafficking		149
Catabolism				
*hemO*	PA0672	Major heme oxygenase	1	10
*bphO*	PA4116	Phytochrome heme oxygenase	1	150
Regulation				
*dnr*	PA0527	NO responsive transcription factor	1	56
*anr*	PA1544	O_2_ responsive transcription factor		56
*prrF1/2*	NA^*b*^	Iron responsive sRNA		35
Synthesis (of HemB)				
*gltX*	PA3134	Glutamyl-tRNA synthetase		151
*hemA*		ALA synthase	NA	1
*hemA*	PA4666	Glu-tRNA reductase		152
*hemL1*	PA3977	Glutamate-1-semialdehyde 2,1-aminomutase		1
*hemL2*	PA4088	Glutamate-1-semialdehyde 2,1-aminomutase		1
*hemL3*	PA5523	Glutamate-1-semialdehyde 2,1-aminomutase	2, 1^*c*^	1
*hemB*	PA5243	PBG synthase		1
*hemC*	PA5260	HMB synthase		1
*hemD*	PA5259	URO synthase		1
*hemE*	PA5034	URO decarboxylase		1
*hemF*	PA0024	Coproporphyrinogen decarboxylase	1	1
*hemN*/*Z*	PA1546	O_2_-independent coproporphyrinogen III oxidase	2	1
*hemG*		Protoporphyrinogen dehydrogenase	NA	1
*hemJ*^*d*^	PA0661	Protoporphyrinogen dehydrogenase	1	1
*hemK*	PA4664	Methyltransferase	1	1
*hemY*	PA5257	Protoporphyrinogen oxygenase		153
*hemH*	PA4655	Protoporphyrin ferrochelatase		1
*hemX*	PA5258	Membrane heme biosynthesis regulatory protein		153
HemD1 synthesis				
*nirF*	PA0516	HemD1 biosynthesis	2	154
*nirL*	PA0514	HemD1 biosynthesis		154
*nirJ*	PA0511	HemD1 biosynthesis	1	154
*nirE*	PA0510	Uroporphyrin III *c*-methyltransferase	1	154
Siroheme				
*cysG*	PA2611	Siroheme synthase		155
*cobA*	PA1778	Uroporphyrin III methyltransferase	1	155
Colbamin (B12)				
*cobI*	PA2904	Cobalt-factor-2 C20-methyltransferase		156
*cobG*	PA2906	Precorrin-3B synthase	1	156
*cobJ*	PA2903	Precorrin-3B C17-methyltransferase		156
*cobM*	PA2948	Cobalt-precorrin-4 C11-methyltransferase		156
*cobF*		Precorrin-6A synthase	NA	156
*cobK*	PA2909	Cobalt-precorrin-6A reductase	1	156
*cobH*	PA2905	Cobalt-precorrin-8 methylmutase		156
*cobB*	PA1273	*c*-Diamide synthase		156
*cobN1*	PA2944	Cobaltochelatase	3	156
*cobN2*	PA1923	Cobaltochelatase		156
*cobO*	PA1272	Cob(I)alamin adenosyltransferase	1^*c*^	156
*cobQ*	PA1277	Adenosylcobyric acid synthase		156
*cobC*	PA1276	Threonine-phosphate decarboxylase		156
*cobD*	PA1275	Adenosylcobinamide-phosphate synthase		156
*cobP*	PA1278	Adenosylcobinamide kinase		156
*cobV*	PA1281	Adenosylcobinamide-GDP ribazoletransferase		156
Hypothetical	PA1280	Alpha-ribazole phosphatase		156
*cobU*	PA1279	Nicotinate-nucleotide-dimethylbenzimidazole phosphoribosyltransferase	1	156
*cobL*	PA2907	Precorrin-6Y C5,15-methyltransferase		156

a Locus for PAO1 strain.

b NA, not applicable.

c Tn insertion disrupts predicted promoter.

d Homology by sequence only.

We observed that the total number of unique Tn insertions decreased sequentially with each enrichment, while a defined subset of insertions increased (Fig. 2E). There was also a noted sharp decrease in the number of unique Tn insertions between the initial unsorted Tn library and E2 populations. Conversely, between E2 and E3 populations, the number of genes with Tn insertions remained more constant. Further analyses using our Tn-Seq analysis software (TSAS) (41) revealed that the sequencing coverage was sufficient for the complexity of our library (Fig. 2F; see Materials and Methods for further details).

**FIG 3:**
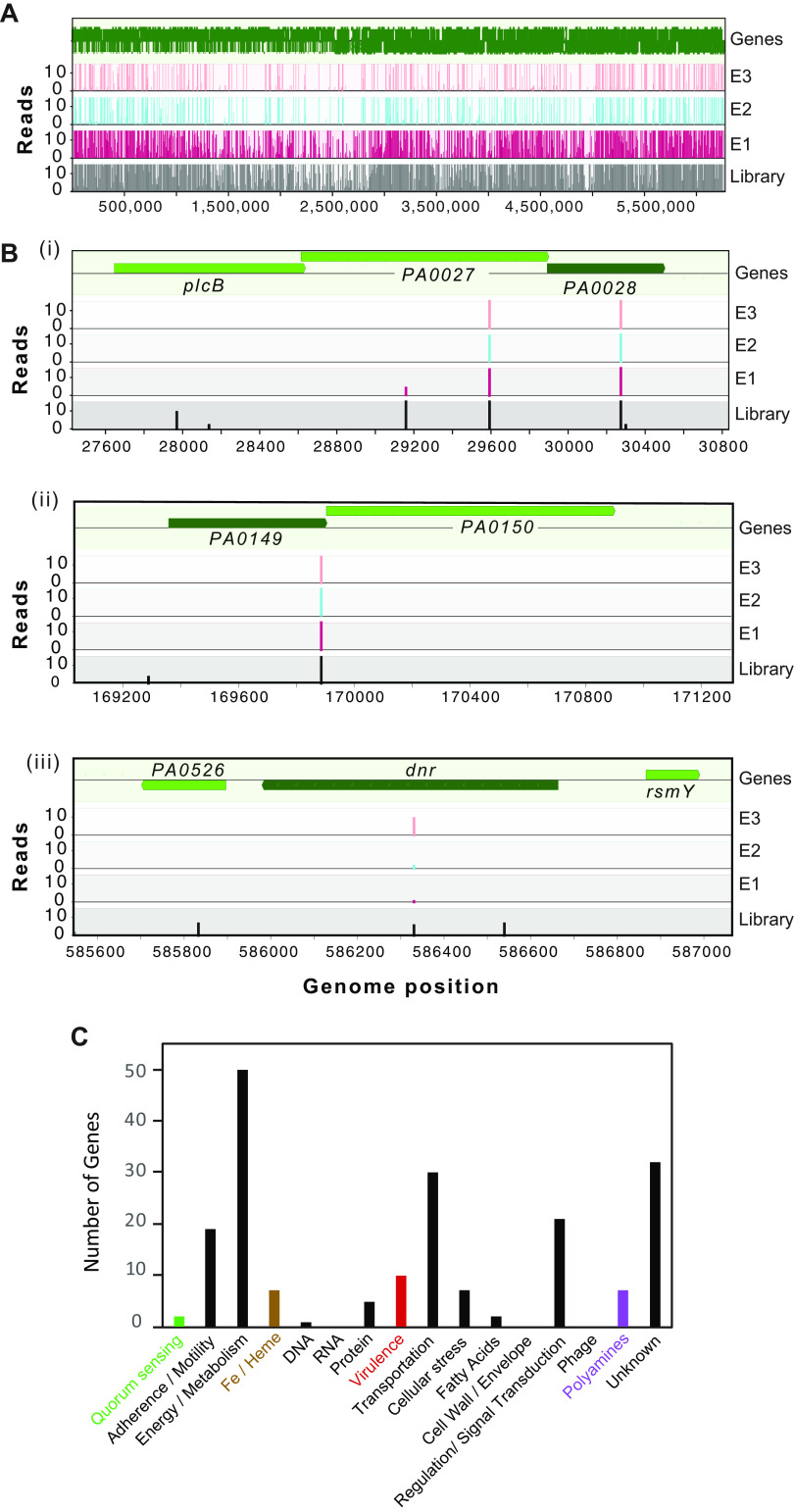
Graphical depiction of Met-Seq Tn insertions and categorization. Graphical displays of Met-Seq Tn insertion profiles of the unsorted MPAO1 library (A) and three of the genes selected for further investigation (B): (i) PA0028, a predicted lipoprotein and part of a three-gene operon shown to encode a zinc-dependent excreted phospholipase system (98), (ii) PA0149, a putative iron-regulated ECF subfamily sigma factor that is 48% identical to pyoverdine regulator PvdS (70, 71), and part of a two-gene operon that includes a FecR homolog (PA0150), and (iii) PA0527, heme biosynthesis regulator Dnr (24, 49). Figures were generated using MochiView (143) graphical outputs. (C) Functional categorization of Tn insertions in the final E3 data set.

The final Met-Seq output (E3 hits) is displayed in Table S1A and contains 188 genes, 150 of which were determined to have a positive change in reads of >2-fold over the initial library representation and 143 of which had a statistically significant *P* value of 0.05 or less. In addition to tabulating these 188 Tn insertions within ORFs, we also applied the same constraints to Tn insertions identified within promoter (regulatory) regions upstream of ORFs (within 100 bp upstream of the ATG start codon; 23 hits) (Table S1B) and, finally, predicted sRNAs and riboswitches based on references (42, 43) (11 hits) (Table S1C). A graphical output showing the entire Tn insertion library coverage of the P. aeruginosa genome is displayed in Fig. 3A, and the enrichment of a subset of Tn insertions within specific genes/operons that were investigated further is shown in Fig. 3B.

10.1128/mSystems.00933-20.8 TABLE S1 Met-Seq enriched genes and statistics. Download Table S1, PDF file, 0.1 MB. Copyright © 2021 Glanville et al. 2021 Glanville et al. This content is distributed under the terms of the Creative Commons Attribution 4.0 International license.

### Assessment of the dim E3 population.

**FIG 4:**
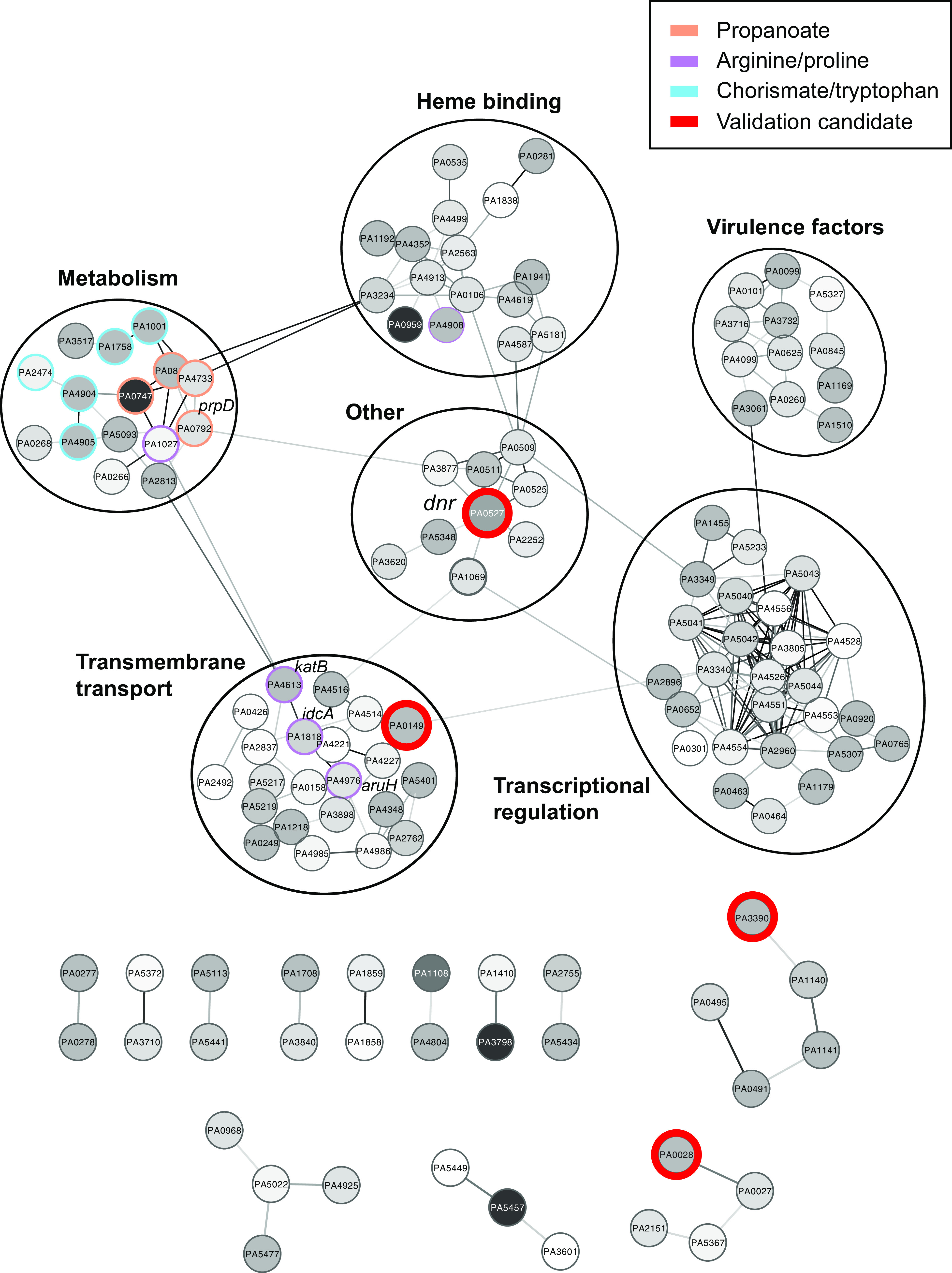
Schematic representation of STRING output and targeted pathway analyses. Lines between genes represent a putative metabolic association. Three pathways involved in arginine/proline, chorismate/tryptophan, and propanoate synthesis are color coordinated purple, cyan, and orange, respectively. Candidates chosen to further validate Met-Seq results (see Fig. 6) are circled in red.

The final dim hits, i.e., ones with Tn insertions presumed to result in less intracellular heme available to the biosensor, were applied to functional categories using manual BLAST searches and also computational analyses via the STRING (44) database. Functional category data generated using STRING displayed with Gene Ontology (GO), KEGG (45), and Cytoscape ontology enrichment analysis (46) are shown in Table S2A to C and graphically in Fig. 4. To expand on these results, we also uploaded Met-Seq hits (Table S1 to C) into the Database for Annotation, Visualization and Integrated Discovery (DAVID) online bioinformatics resource, (47), which resulted in an expanded list of 15 functional clusters of genes and further categorized Met-Seq hits into (i) heme-related, (ii) iron-related, (iii) cytochrome and electron transport-related, and (iv) siderophore-related functional categories (Table S2D).

10.1128/mSystems.00933-20.9 TABLE S2 Computational outputs. STRING and DAVID annotation is explained in references 44 and 47. Download Table S2, PDF file, 0.1 MB. Copyright © 2021 Glanville et al. 2021 Glanville et al. This content is distributed under the terms of the Creative Commons Attribution 4.0 International license.

**FIG 5:**
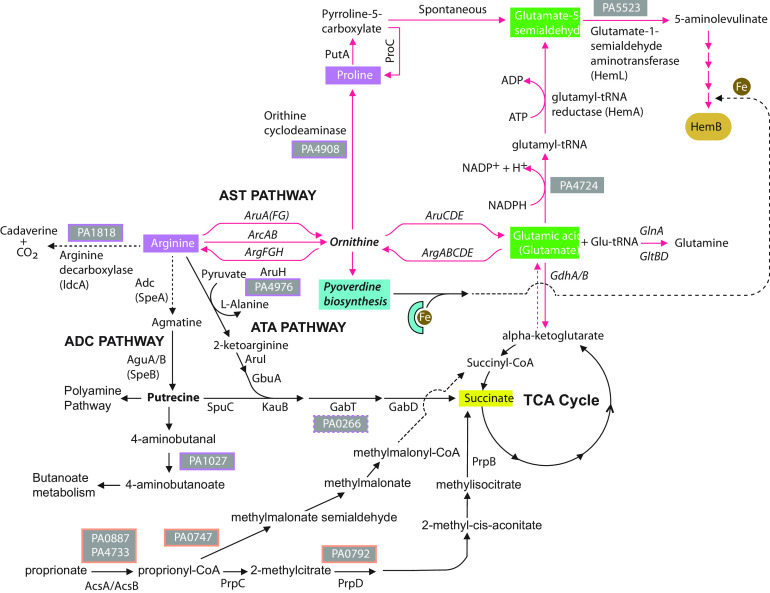
Diagram of Met-Seq Tn insertion candidates derived from STRING output of metabolic pathways that lead to C_5_ heme biosynthesis. Genes identified by Met-Seq are displayed as their locus numbers in boxes (purple boxes are proline/arginine and orange boxes are propanoate metabolism-related Tn insertions). Data demonstrate that disruptions in metabolic flux through the preferred TCA carbon source of P. aeruginosa, succinate (50), or alternatively, the proline/glutamic acid precursor ornithine, result in HemB synthesis disruption and would therefore result in a “darker” population in the Met-Seq screen.

STRING-generated data identified metabolic connections within 6 major categories that consisted of (i) heme-binding proteins, (ii) transmembrane/transport, (iii) metabolism, (iv) virulence factors, (v) transcriptional regulation, and (vi) “other.” Interestingly, the genes identified in the “other” category made centralized connections to the remaining five categories and included Dnr, a transcription factor which binds heme and regulates heme biosynthesis and anaerobic metabolic adaptation in pseudomonads and other Gram-negative bacteria (24, 48, 49) (Fig. 4). Of particular interest were the metabolism-related hits, which collectively pointed to the utilization of specific amino acids and other molecular building blocks required to synthesize the TCA intermediate succinate, a preferred carbon source of P. aeruginosa (50). Succinate would then be predicted to drive heme biosynthesis (Fig. 5).

In total, the genes of the most represented functional category identified by manual annotation in the E3 population were involved in metabolism, followed by genes of unknown function, transport, regulation, and adherence/motility (Fig. 3C). Notably, Tn enrichments were observed within two different arabinose transport systems (PA5219 and PA4113) (Table S1A), which served as excellent internal controls, as we used arabinose to induce biosensor expression.

A comprehensive diagram of heme-related pathways identified by Met-Seq is shown in Fig. S4. Below, we describe noteworthy Met-Seq hits (Table S1A to C) in five functional categories related to (i) heme biosynthesis and uptake, (ii) siderophores and iron, (iii) central metabolism, (iv) virulence, and (v) unknown function.

10.1128/mSystems.00933-20.4 FIG S4 Diagram showing potential heme metabolic associations from Met-Seq data. Boxes around gene annotations are representative of the following functional categories. Brown boxes, signal transduction; green, metabolic functions; red, virulence factors; grey, genes encoding proteins found in OMVs. The solid box at the bottom right represents outside the bacterial cell, whereas the rest of the schematic diagram represents inside the cell. Dotted red lines represent the lipid-to-beta oxidation-to-glyoxylate shunt metabolic flux, whereas purple lines represent entry points into C_4_ or C_5_ heme biosynthesis pathways. Blue dotted lines represent the choline/glycine/betaine route to heme synthesis via the C_4_ pathway. Arrows represent the direction of the metabolic pathway. Download FIG S4, EPS file, 1.8 MB. Copyright © 2021 Glanville et al. 2021 Glanville et al. This content is distributed under the terms of the Creative Commons Attribution 4.0 International license.

### (i) Heme biosynthesis and uptake.

ALA is the first committed precursor for heme biosynthesis and can be made via either the C_4_ or C_5_ pathway (1). In the C_5_ pathway, HemA initially reduces glutamyl-tRNA^glu^ to glutamate-1-semialdehyde, which is subsequently converted to ALA by HemL (glutamate-1-semialdehyde aminotransferase [1]), and is the sole known synthesis pathway for most prokaryotes, including pseudomonads (1, 51). The alternative pathway is the C_4_ or Shemin pathway, which generates ALA instead by the condensation of succinyl-CoA and glycine through the action of ALA synthetase (Fig. S4), and is predominantly found in eukaryotes and some select bacteria (1, 51).

An interesting gene identified by Met-Seq that could contribute to C_5_ heme synthesis was glutamyl queuosine-tRNA^Asp^ synthetase (GluQ-RS) (locus number PA4724; reads enriched 4-fold) (Table S1A). GluQ-RS is a paralog of the canonical glutamyl-tRNA synthetase catalytic domain, responsible for catalyzing the formation of glutamyl-queuosine on the wobble position of tRNA^Asp^ (52). Although a function has not been determined for the accumulation of glutamyl-queuosine other than a possible role in stress response signaling (53), its function could potentially alter the overall glutamate pool required for the C_5_ heme biosynthesis pathway. Genes that could affect synthesis of ALA itself were also found in the E3 population, including a potential HemL paralog, PA5523, whose precise metabolic role remains to be determined.

**FIG 6:**
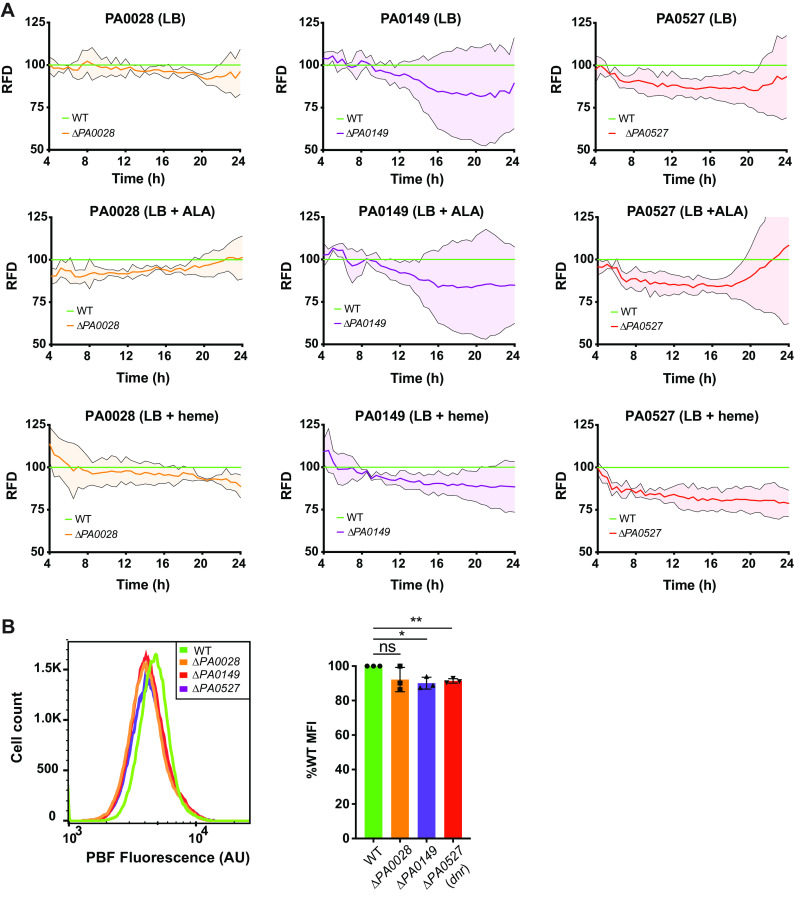
Assessment of biosensor activity in mutants. (A) Cultures of WT MPAO1 and deletion mutants harboring pIFPHO were grown in either LB, LB plus 50 μg/ml ALA, or LB plus 5 μM hemin. Biosensor expression was induced with the addition of 0.2% arabinose. Absorbance (600 nm) and PBF fluorescence (excitation 675 nm, emission 725 nm) were measured every 30 min for 24 h. The mean fluorescence densities from three biological repeats for PA0028 (orange line), PA0149 (purple line), and *dnr*/PA0527 (red line) are expressed as percentages of the mean fluorescence density of the WT. RFD, relative fluorescence density. Error bars represent SDs and are depicted above and below the normalized mean lines continuously in a lighter color. (B) Flow cytometric analysis of biosensor-expressing mutants after 14 h of growth in M-56 medium. Cells were fixed with 3% PFA before analysis by flow cytometry. (Left) Representative histogram of PBF fluorescence in each strain. (Right) The mean median fluorescence intensity (MFI) values from three biological repeats are expressed as percentages of the MFI of WT MPAO1. Error bars represent SDs. *, *P* < 0.05; **, *P* < 0.01; ns, not significant by a one-sample *t* test with Wilcoxon test.

We also identified two enriched Tn insertions within the heme *d*_1_ biosynthesis pathway operon (*nirJ* [*PA0511*] and *nirL* [*PA0509*], enriched ∼3.6- and 2-fold, respectively) (Table S1A). Heme *d*_1_ is a specific cofactor used by dissimilatory nitrite reductase (a key enzyme in the denitrification pathway), which occurs under anaerobic or low-oxygen conditions and allows *Pseudomonas* sp. to utilize N-oxides as terminal electron acceptors in low-oxygen environments such as the cystic fibrosis lung (54). The importance of denitrification in influencing P. aeruginosa heme levels was further supported by an insertion in a possible *norV* homolog (*PA4348*, enriched >4-fold) and *norD* (*PA0525*, enriched 1.5-fold), both involved in nitric oxide detoxification (55) (Table S1A). The *nir* denitrification operon has been directly tied to iron/heme regulation by way of the sRNA *prrF1*/*2-prrH*, as well as the nitric oxide-sensing and heme-binding transcription factor Dnr (50, 56) that directly controls *hemA* expression and therefore heme biosynthesis (24). In support of this regulatory relationship, the *dnr* Tn insertion clone was enriched in our E3 data set (enriched 6.4-fold) (Fig. 3B and Table S1A) along with the gene encoding the integration host factor (IHF) regulator histone-like binding protein (*PA5348*, enriched >2-fold), which has also been shown to directly regulate the *hemA* transcript (24). A clean deletion in the *dnr* gene (*PA0527*) and measurement of biosensor fluorescence density in the presence of either ALA or heme indeed demonstrated a reduction in reporter signal (Fig. 6A and B).

**FIG 7:**
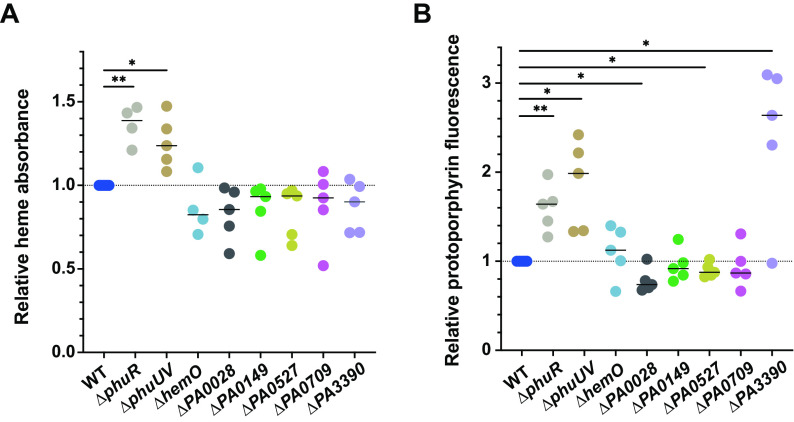
Intracellular heme measurements. (A) Hemochrome assay. Absorbance-based measurement of WT MPAO1 or MPAO1 with clean deletions (not harboring the biosensor) of *phuUV*, *phuR*, *hemO*, *PA0028*, *PA0149*, *dnr* (*PA0527*), and ABM domain genes *PA0709* and *PA3390* following 14 h of growth in M9 plus 5 μM heme. Cells were lysed, and heme levels were determined by pyridine hemochrome and expressed relative to WT (57). (B) Fluorescent heme assay. Cells were grown as described for panel A, and the heme levels were determined as described in the legend for Fig. 1G and in Materials and Methods (30). The horizontal lines indicate the medians from five biological repeats. Individual data points are plotted. Statistically significant differences were determined using a one-sample *t* test with Wilcoxon test. *, *P* < 0.05. **, *P* < 0.01.

To further verify that Met-Seq had provided a gene set that influenced actual intracellular heme levels, we also measured intracellular heme directly and, this time, in the absence of the biosensor, using both a hemochrome assay based on absorbance (57) and a fluorescence-based assay that ultimately detects protoporphyrin (30) (Fig. 7A and B). When heme levels were measured in the *dnr* mutant (Δ*PA0527*) and compared to those in the WT, we observed a concomitant reduction in total intracellular heme (Fig. 7), verifying that Dnr positively influences heme levels in P. aeruginosa (24). Although the Δ*phuR* control showed higher heme concentrations, which paralleled our results with the biosensor present in this strain, interestingly, the Δ*phuUV* and Δ*hemO* strains trended differently, with the Δ*phuUV* strain (in the absence of the biosensor) showing increased heme levels and the Δ*hemO* strain trending slightly lower or unchanged depending on the assay (compare Fig. 1 and Fig. 7). We again attribute this result to the biosensor having an effect on intracellular heme levels in some of the mutants.

In general, Met-Seq failed to identify enrichment of Tn insertions within most known heme uptake systems (e.g., *phuSTUVW* operon), even though Tn insertions were present within most of these genes in the initial library (Table 1). Although some were enriched within the first round (E1) (e.g., *phuV*), the reason for this remains unclear but could be due to these mutants being outcompeted during the initial library heme starvation step and/or subsequent growth and enrichments. Nevertheless, STRING output identified 16 known heme-binding proteins from our Met-Seq screen.

PsdR was one protein identified by STRING as having a connection to heme-binding proteins (PA4499; enriched by ∼2-fold) (Table S1 and S2A to C; Fig. 4). PsdR is a transcription factor which functions to repress the *dpp* transporter operon, which is responsible for uptake of peptides in P. aeruginosa (58, 59). Interestingly, the *dpp* transporter operon is induced under low-iron conditions and involved in the binding and uptake of heme as well as the heme precursor ALA in many other Gram-negative pathogens, including Salmonella enterica serovar Typhimurium (60), Escherichia coli (61), and Haemophilus influenzae (62), but thus far not P. aeruginosa. This observation suggests a link between intracellular heme levels and regulation of *dpp*, and that the *Pseudomonas* sp. Dpp homolog could also serve as a heme and/or ALA transporter.

Another interesting tetrapyrrole-related hit was identified as a predicted cobalamin (vitamin B_12_) synthesis pathway riboswitch, *cobG* (42, 63) (between the *cobG* open reading frame [*PA2906*] and *PA2907*; enriched by 4-fold). This *cobG* riboswitch homolog was identified in a P. aeruginosa transcriptome sequencing (RNA-seq) study (43) and contains the signature sequences known to bind cobalamin (64), a metabolite whose synthesis directly branches from the heme intermediate product uroporphyrinogen III (Fig. S4). Future work will determine if this sRNA is a novel riboswitch that regulates cobalamin and heme biosynthesis pathways in this pathogen.

### (ii) Siderophore and iron-related genes.

Manual (Table S1) and computational (Table S2) annotation identified several enriched Tn insertions within genes that govern iron acquisition through siderophore biosynthesis and uptake. These data suggest a regulatory connection between cellular heme levels, its biosynthesis, and iron uptake systems. Notable hits included *chtA* (*PA4675*) (65) and *fptA* (*PA4221*) (Fig. S5A), both of which have been identified in outer membrane vesicles (OMVs), the genes encoding the master pyochelin regulator PchR (*PA4227*) (66, 67), the PiuA iron receptor (*PA4514*) (68), and the predicted FoxB xenosiderophore operon (*PA2464*) (69). Two genes encoding ECF factors, *PA1363* and *PA0149*, predicted to regulate siderophore and metal uptake, respectively, also emerged in our E3 population (*PA0149* was enriched 4-fold) (Table S1A).

10.1128/mSystems.00933-20.5 FIG S5 MochiView-generated diagrams of Met-Seq enrichments of key genes mentioned in the text. (A) Genes or operons of (i) the *creBC* two-component signaling system (77), (ii) the *vanAB* vanillate synthesis operon, (iii) the *phnAB* anthranilate (PQS) biosynthesis operon, (iv) the *tseF* T6SS effector, (v) the *fptA* siderophore transporter gene, and (vi) the iron/siderophore associated ECF sigma factor PA1363. Note that neither *fptA* or *tseF* Tn insertion enrichments were included in Table S2, as they did not meet the minimal read cutoff. Arrows indicate emerging reads of less than 10 in the E3 population. The E3 sort is highlighted in orange. (B) MochiView graphic diagram of the predicted PA3577 riboswitch Tn insertion site and annotated 3-dimensional RNA structure prediction. The Tn insertion is colored red, the ribosome binding site (RBS) is yellow, and the start codon (ATG) is green. The structural model was generated using RNAComposer (http://rnacomposer.cs.put.poznan.pl) and displayed using MacPyMOL. Download FIG S5, EPS file, 1.9 MB. Copyright © 2021 Glanville et al. 2021 Glanville et al. This content is distributed under the terms of the Creative Commons Attribution 4.0 International license.

Interestingly, PA0149 bears 48% identity to pyoverdine regulator PvdS (70, 71) and is in a predicted operon containing a homolog of the iron uptake regulator FecR (PA0150) (Fig. 3B) (70, 72). Similar to that for Dnr, we made a clean deletion of *PA0149* in MPAO1, introduced the biosensor, measured fluorescence density over time with either ALA or heme added, and also measured total intracellular heme levels using two different methods. Results demonstrated a lower biosensor florescence level over time compared to that for the WT (up to 22% reduction) (Fig. 6A and B). In the absence of the biosensor, more direct measurements of the heme levels in the mutant showed a similar reduction compared to the WT level (Fig. 7), indicating that PA0149 is linked to intracellular heme levels and that Met-Seq was capable of identifying such novel associations.

Enriched Tn insertions were also observed within several sulfur transport and metabolism genes, which have been explicitly linked to pyochelin biosynthesis through replenishing the cysteine pool in pseudomonads and related species (73, 74) (Fig. S4). These insertions were found within the sulfite reductase gene *cysI* (*PA1838*) and other genes involved in sulfate transport (*cysW* [*PA0281*], *PA0278*, and *PA2563*) (Table S1A). *cysW* and *PA0278* Tn insertions were enriched 4-fold in the E3 population (Table S1A), suggesting potential importance in heme regulation. In further support of a connection between sulfur and heme/iron metabolism, Nelson et al. have reported that cysteine biosynthesis and sulfur assimilation pathway protein levels are associated with intracellular iron depletion (38). Additionally, l-cysteine can be catabolized to glutamate, which then can enter the C_5_ heme biosynthesis pathway directly (Fig. S4).

Another enriched Tn insertion related to iron acquisition was found within the upstream regulatory region of the *phzH* ORF, an enzyme that converts the chorismate-based metabolite phenazine-1-carboxylic acid (PCA) to phenazine-1-carboxamide (75) (Fig. S4). In the absence of siderophores, PCA is able to reduce ferric iron (Fe^3+^) to ferrous iron (Fe^2+^), enabling iron acquisition (76). In addition, a Tn insertion was identified downstream of a PhzF1 homolog ORF encoded by *PA3578* (enriched 2.5-fold) (Table S1C; Fig. S5). This region is predicted to be transcribed (42, 43) and encompasses the 105 bp upstream of the small hypothetical gene *PA3577*. As illustrated in Fig. S5B, the potential leader RNA is predicted to form a hairpin loop secondary structure and could therefore encode a novel *PA3577*-associated riboswitch.

### (iii) Central metabolism.

The greatest number of heme-related genes identified by both manual and computational Met-Seq analyses were associated with the regulation and enzymatic activity of central metabolic pathways (Fig. 3C). One example is *creB* (*PA0463*) and *creC* (*PA0464*) (Table S1; Fig. S5A), a two-component signaling system that regulates production of the TCA cycle entry molecule acetyl-CoA (77). Interestingly, the CreC histidine kinase recognizes a peptide derived from the iron-binding host protein lactoferrin, which is an important source of iron during infection (78). In this same pathway, Met-Seq identified insertions both in *acsA* (*PA0087*) and *acsB* (*PA4733*), the aspartate decarboxylase *panD* (79) (enriched by >33-fold) (Table S1B), and finally, several genes linked to beta oxidation, all of which are involved in the formation of acetyl-CoA, which ultimately drives the TCA cycle and therefore heme production (Fig. S4). Finally, the second most enriched Tn insertion was within *PA0747* (enriched by >41-fold) (Table S1A) and is part of a predicted 5-gene operon (*PA0743* to *PA0747*) required for P. aeruginosa virulence and siderophore production (80). Although the precise function of PA0747 is unknown, it bears a 47% identity and predicted structural similarity to MmsA, an enzyme that uses CoA and valine to eventually synthesize succinyl-CoA from the propionate pathway (81) (Fig. S4). Of note, the propionate pathway was identified as enriched by STRING (Fig. 4 and 5) and points to propionate as a carbon source that P. aeruginosa might use to feed into the TCA cycle to enhance heme production.

One of the most prominently represented metabolic pathways identified from our screen was that of chorismate (Fig. S4), a central metabolite best recognized as the precursor for aromatic amino acids (82) and the pseudomonas quorum sensing (PQS) molecule. PQS can either be synthesized via the precursor anthranilate or by catabolism of tryptophan via the kynurenine pathway (82, 83) (Fig. S4). Met-Seq identified enrichments in the *phnA*/*B* operon responsible for anthranilate synthesis (*PA1001*/*PA1002*) (Fig. S5A) and a gene encoding a predicted kynurenine aminotransferase, whose precise function remains to be determined (84) (*PA3798*; enriched by >41-fold) (Table S1A). In addition to PQS, several other metabolic pathways connected with that of chorismate were identified in the screen, including that of (i) the siderophore pyochelin (PchR; Tn insertion in *PA4227*), (ii) phenazine biosynthesis (PhzH; Tn insertion within the promoter region of *PA0051*) (Table S1B), (iii) folate biosynthesis via the PabABC pathway (Tn insertion in *pabB* [*PA1758*]), and several within the *AroBCDEFG* operon (*aroG1* [*PA1750*], *aroG2* [*PA2843*], and again PhzH [*PA0051*]) (85) (Fig. S4; Table S1A to C).

Also noteworthy, Met-Seq identified two enriched Tn insertions within the vanillin synthesis operon *vanAB* (*PA4904*/*PA4905*; both enriched >4-fold) (Fig. S5A), responsible for the synthesis of this aromatic metabolite that branches off the shikimic acid pathway (Fig. S4). The VanAB proteins together comprise active vanillate demethylase, an enzyme required for the conversion of vanillin to 3,4-dihydroxybenzoic acid (3,4-DB). 3,4-DB is a known microbial siderophore and possesses the iron-binding moiety of petrobactin (86, 87), a siderophore which was not previously described in P. aeruginosa. In other Gram negatives, oxidized vanillin (vanillic acid) regulates quorum sensing, biofilm formation, virulence, and, importantly, iron transport and heme biosynthesis (88) by inhibition of the fatty acid synthesis protein FabG, a homolog of which is also enriched in our Met-Seq data by >4-fold enrichment (PA0182) (Table S1A).

STRING analysis of our E3 Tn insertion population revealed additional metabolic pathways that were not obvious to us from manual annotation (Fig. 4). Especially interesting were arginine and proline pathways, which are metabolically linked (Fig. 4 and 5). Routes to heme biosynthesis through arginine include both arginine succinyl transferase (AST) and arginine transaminase (ATA) pathways (89) or, alternatively, arginine can be catabolized either through the arginine decarboxylase (ADC) pathway to the polyamine entry molecule putrescine or by the arginine decarboxylase IdcA to cadaverine (Fig. 5). The ADC and ATA pathways both provide precursors for the TCA entry substrate succinate, a preferred carbon source of P. aeruginosa, whereas the AST pathway eventually produces the intermediate ornithine. From this central point, ornithine can be converted to either glutamic acid, which can enter the heme biosynthesis pathway at the HemA stage, or proline, which can enter at the HemL stage (Fig. 5). Enriched Tn insertions were obtained by Met-Seq in all the aforementioned pathways with the exception of the AST route.

### (iv) Virulence factors.

Our Met-Seq screen identified a surprising number of virulence factors (Fig. S4), a functional category that, to the best of our knowledge, was not previously suggested to regulate intracellular heme levels. Met-Seq hits in this category included PopB, the main chaperone for type III secretion system (T3SS) effectors (90) (enriched >4-fold). However, virulence factors associated with the type 6 secretion system (T6SS) were much better represented (91). These hits included Tn insertions within genes encoding a predicted lipase with an α-β hydrolase domain (*PA0260*; 2-fold enrichment) (92), the Tle4 phospholipase family protein T6SS effector TplE (*PA1510*; 4-fold enrichment) (93), and the Vrgb1-dependent nuclease toxin (94) (*PA0099*, 4-fold enrichment). Several other lipases and host lipid-degrading virulence factors were also enriched, including SphC and CerN that are both important for host lung surfactant sphingolipid degradation (95), the secreted arachidonate 15-lipoxygenase (LoxA) that helps limit host-induced inflammation (96), and two predicted patatin-like domain-containing lipases (the *PA2660/PA2661* operon and RssA [PA3241]) (97). Finally, the *PA0026-PA0028* operon was enriched in our screen, which is involved in lipid chemotaxis and excretion of a phospholipase (98). Subsequent deletion of *PA0028*, a gene of unknown function in this operon, was then reassessed using our biosensor. Results trended toward a decrease in biosensor signal that varied (up to an 11% decrease) depending on the growth phase and addition of ALA or heme (Fig. 6A and B). Subsequent measurements comparing intracellular heme levels between WT MPAO1 cells and the Δ*PA0028* strain showed a decrease in total intracellular heme concentrations, further verifying that this operon influences intracellular heme levels (Fig. 7).

After the *PA0026-PA0028* operon facilitates taxis and enzymatic breakdown of phosphatidylcholine (PC) to choline, the choline can be internalized and further catabolized. Indeed, Met-Seq also identified two homologs of the choline dehydrogenase BetA (PA3710 and PA5372), which metabolizes choline to glycine betaine (GB). GB then eventually yields glycine, which can then enter the C_4_ heme biosynthesis pathway directly (Fig. S4). Intriguingly, both choline and GB can act as sources of energy to promote survival in the lung and have been shown to regulate hemolytic phospholipase C (PlcH) production in *Pseudomonas* sp. (99–101). Further support of a possible phospholipid-heme regulatory connection illuminated by our studies comes from several enriched genes that are able to transport choline and/or GB into the bacterial cell (presumably after extracellular host phospholipid cleavage). These Met-Seq hits included (i) *PA5378* (*cbcX*), which encodes a periplasmic choline-binding protein and is part of the *cbcXWV* choline uptake operon (102), (ii) *PA5401*, which encodes a predicted GB transporter, and (iii) two genes encoding predicted glycine transporters (*PA2252* and *PA3641*) (Table S1A and Fig. S4). Taken together, the plethora of Tn insertions identified by Met-Seq within the GB/choline uptake and catabolism genes are consistent with the literature, which has clearly shown that both GB and choline are potent stimulators of heme biosynthesis and B_12_ biosynthesis in pseudomonads (103–105). Nevertheless, as pseudomonads and most other nonphotosynthetic bacteria do not have the C_4_ biosynthesis pathway, which relies on glycine as an entry substrate for heme synthesis (Fig. S4), the question still remains as to the precise metabolic roles choline/GB play in heme regulation.

### (v) Unknown function.

**FIG 8:**
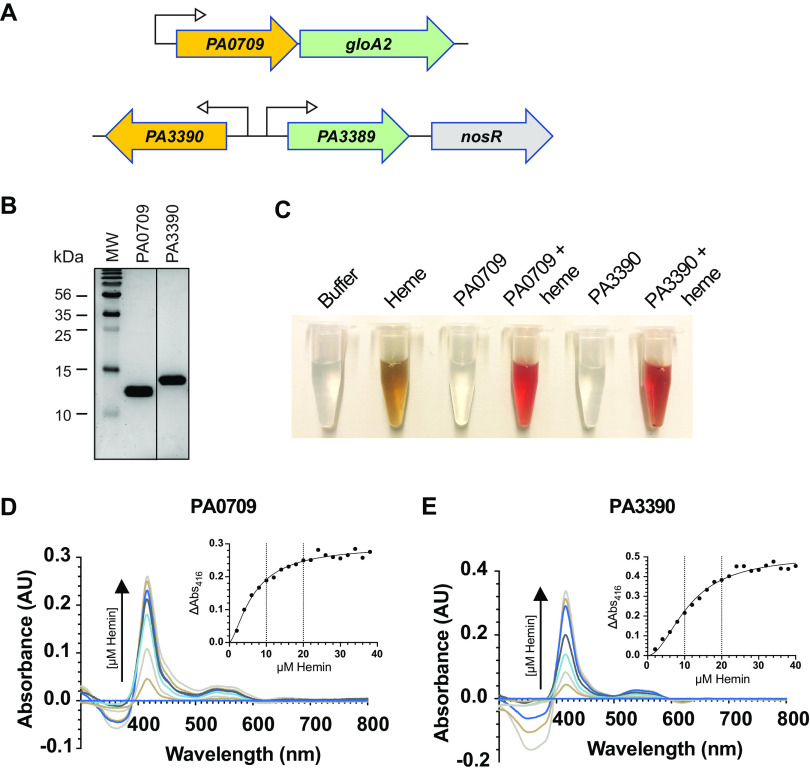
Heme binding of PA0709 and PA3390 protein. (A) Schematic representation of *PA0709* and *PA3390* ABM domains and surrounding genome locations in the P. aeruginosa PAO1 chromosome. *PA0709* shares an operon with the glyoxal detoxification enzyme GloA2, and *PA3390* shares a divergent promoter with a gene encoding another predicted glyoxalase (a lactoylglutathione lyase) detoxification enzyme, PA3389. *nosR* is the first gene in the nitrous (Nos) denitrification operon. (B) SDS-PAGE gel of 5 μg of purified protein used in these assays. (C) Dramatic color change when 100 μM heme is added to 10 μM PA3390 or PA0709 protein, suggestive of heme binding to the protein. Absorbance spectra of PA0709 (D) and PA3390 (E) pure protein with increasing heme concentrations and (inset) peak absorbances at 412 nm. Based on these results, PA0709 is predicted to bind one heme molecule per ABM domain, and PA3390 is predicted to bind two heme molecules per ABM domain. Data are representative of three biological repeats.

Some of the most exciting findings from these studies were genes of unknown function. To validate that such genes were indeed a contributing factor to heme biosynthesis and not artefactual, we chose two related genes to further investigate from our E3 population, *PA3390* and *PA0709*, which we first determined influenced actual intracellular heme levels (Fig. 7). Both of these genes are ABM domains, some of which have been implicated in the catabolism of heme in Staphylococcus aureus (106), Mycobacterium tuberculosis (107), and many other microbes (108). Interestingly, both *PA3390* and *PA0709* are transcriptionally linked to glyoxal detoxification enzymes (109, 110), possibly suggesting a functional connection to the presence of this ubiquitous toxin (Fig. 8A). Deletion of *PA0709* and *PA3390* resulted in a reduction in the total cellular heme levels as compared to WT using the absorption assay. Using the fluorescence-based assay, the *PA0709* deletion strain trended similarly, whereas the *PA3390* deletion instead resulted in a large and statistically significant increase in heme levels (Fig. 7). The reason for this increase in heme levels in the *PA3390* deletion strain remains unclear but could be attributed to the assay itself, where the iron is stripped from heme and protoporphyrin IX fluorescence is ultimately measured. These data indicate that although PA0709 and PA3390 share much homology, they could play different cellular roles in heme homeostasis.

As ABM domains have been shown to bind and metabolize heme, the next logical experiment was to investigate if PA0709 and PA3390 could also bind heme. To this end, both PA0709 and PA3390 were expressed and purified to homogeneity (Fig. 8B), and heme was added incrementally before absorbance spectra were collected. After heme addition, the samples exhibited a notable change in color (Fig. 8C) and the typical increase in absorbance (peak 412 nm) indicative of protein-heme interactions (Fig. 8D and E). An examination of the saturation concentrations suggested that PA0709 binds one molecule of heme per monomer, whereas PA3390 binds two (similar to the heme-degrading enzyme MhuD [107]). Next, we examined if PA0709 and PA3390 were heme-degrading enzymes by first saturating the proteins with heme and then adding an electron donor and monitoring absorbance over time. Results shown in Fig. S6 showed no appreciable decrease in absorbance at 412 nm and no increase in absorbance around 575 nm, which is indicative of biliverdin accumulation (107). These data demonstrate that both ABM domains do not degrade heme and likely have another regulatory function, such as signaling or chaperoning.

10.1128/mSystems.00933-20.6 FIG S6 Heme degradation assay. Absorbance spectra of *holo* PA0709 and PA3390 over time after addition of ascorbic acid, or not, to determine if the proteins could catabolize heme. The assay was carried out in the presence of catalase as described by A. Chao and C. W. Goulding CW, Biochemistry 58:489–492, 2019, https://doi.org/10.1021/acs.biochem.8b01198. Download FIG S6, EPS file, 0.7 MB. Copyright © 2021 Glanville et al. 2021 Glanville et al. This content is distributed under the terms of the Creative Commons Attribution 4.0 International license.

## DISCUSSION

The ability to exploit biosensors to more directly monitor intracellular metabolites provides the most accurate assessment of intracellular metabolite concentration in real time. Here, we describe a method (Met-Seq) where we combine a heme biosensor with FACS and extend the available Tn-Seq technology to identify new factors that influence the intracellular heme concentrations in the pathogen P. aeruginosa. The uniqueness of Met-Seq over other massively parallel sequencing methods is that it can be used to monitor metabolites directly, rather than relying on inference through measurement of promoter activity, RNA (RNA-seq), or protein levels (proteomics), all of which do not always correlate with concentrations of their metabolic end products due to posttranscriptional and posttranslational regulation (111).

Aside from the more obvious tetrapyrrole-related genes obtained in the screen, a less understood and more complex overall picture emerged of P. aeruginosa intracellular heme control (collectively illustrated in Fig. S4 in the supplemental material and represented by STRING output data in Fig. 4). One major metabolic pathway that appeared to surface extensively was the chorismate pathway (relevant Met-Seq hits highlighted in cyan in Fig. 4). Chorismate is a precursor molecule of the three aromatic amino acids and many other important aromatic compounds (e.g., for the synthesis of the siderophore enterobactin, folate, ubiquinones, and the quorum sensing signal PQS) (Fig. S4). We detected one or more enriched Tn insertions within all of these pathways, including three insertions within the Shikimate pathway, responsible for producing chorismate itself (112). The precise reason why this central metabolic pathway was substantially enriched in our screen remains unclear; however, we can speculate as to some possibilities. In a direct connection to iron regulation, chorismate is the precursor for PQS, which has been shown to directly bind iron and aid in its uptake through the siderophore pyochelin and its transporter FptA (enriched in the E3 population) (Table S1A). This process is facilitated by the formation of OMVs through the action of the T6SS and the effector TseF (PA2374, also enriched in our screen) (Fig. S5A) (113). TseF is incorporated into the PQS-containing OMVs and interacts directly with the PQS-pyochelin complex to then facilitate internalization of the PQS-iron through FptA and the OprF porin (113) (Fig. S4). Indeed, it is PQS itself that is responsible for driving the formation of OMVs (114).

A possible explanation for a regulatory connection between T6SS effectors and intracellular heme/iron levels could involve OMVs, where they help deliver virulence factors (e.g., hemolysins) into host cells to acquire nutrients during infection, especially iron and heme (115, 116). Such action is generally thought to result from stress brought on by the competitive, nutrient-deprived environment of the host (117) (or in this case, possibly, the minimal medium used in the Met-Seq screen). In support of this connection, in addition to the TseF effector, we found a total of 10 genes with enriched Tn insertions in our E3 population that have all been identified as present in OMVs (118) (boxed in gray in Fig. S4). Moreover, studies have shown that nutrient-driven stresses cause a hyperproducing OMV phenotype (119), which likely results in virulence, damage to host tissue, and the coordinated “theft” of host nutrients. An intriguing hypothesis is that a direct regulatory connection exists between iron/heme acquisition, siderophore and heme biosynthesis, and coordination of OMVs and their associated virulence factors, all processes known to be directed by the chorismate-derived PQS quorum sensing signal (113, 120).

A possible connection between virulence factors and heme biosynthesis control is logical. As heme is an essential metabolite, bacterial cells must tightly regulate the synthesis of heme, a process that is more energetically costly than simply acquiring it from the host (121). Indeed, iron and heme can be readily extracted from certain tissues with ease; therefore, where heme is abundant, it is conceivable that P. aeruginosa ceases *de novo* biosynthesis while obtaining this essential nutrient by excreting virulence factors such as hemolysins and upregulating heme transport and its associated processivity systems. In this work, we observed such a connection *in vitro*, where the addition of extracellular heme lowered biosensor fluorescence (Fig. S2D to F) and therefore, by association, is suggestive of a decrease in intracellular heme biosynthesis when heme is plentiful in the extracellular milieu. Indeed, this phenomenon has been known to exist in E. coli for decades (32). A further expansion on this hypothesis that involves *in vivo* host-pathogen interactions comes from a recent report which shows a direct regulatory link between cholera toxin expression and iron/heme acquisition in the host (122).

In addition to the internalization of heme itself to be used as a source of iron, or as a cofactor to be directly incorporated into the cell’s metabolic processes, pathogens might also internalize other host nutrients, such as certain amino acids, to then feed into the heme biosynthesis pathways. In this regard, Met-Seq identified Tn insertions within several genes in such metabolic pathways that affected internal heme levels. For example, it is well established that catabolism of arginine and proline results in metabolites that can then enter the TCA cycle (89, 123, 124) and C_5_ pathway directly to produce heme, respectively. Computational methods using our data indeed revealed several genes involved in the breakdown of these amino acids that had been enriched during the Met-Seq process (Fig. 4 and 5). These data would indicate that arginine and proline could be important precursors for synthesizing heme. In particular, arginine catabolism was blocked through Tn insertions within the IdcA decarboxylase and also within two pathways that feed into the TCA cycle (Fig. 5). However, we found no enriched insertions within the AST pathway that results in ornithine and the eventual production of glutamic acid, a metabolic requirement for HemA enzymatic activity (Fig. 5). Because the original library contained insertions within the AST pathway, these data point to a possible metabolic preference for P. aeruginosa to use arginine and polyamines as a means to feed the TCA cycle at the succinate entry point in order to expedite heme biosynthesis. Since amino acids and succinate are preferred sources of carbon utilization for this pathogen (50), these data would support this hypothesis. In further support, a recent proteomics study in P. aeruginosa indeed revealed that iron starvation results in a repression of arginine biosynthesis through the same ornithine intermediate in a *prrF*-dependent manner (Fig. 1) (35, 38). Ornithine is not only a precursor for succinate but also a precursor for the synthesis of the important siderophore pyoverdine required for iron uptake in OMVs. Taken together, these data point to ornithine as a possible central hub in the coordination of iron acquisition and heme biosynthesis, whose activity might be governed by the available concentrations of arginine and proline.

One of the major categories of protein function identified by STRING was heme-binding proteins (Fig. 4; Table S2A to D). Since a large category of identified genes were of unknown function, we wanted to explore if some of these proteins also could bind heme and were involved in its metabolism. Met-Seq hits PA0709 and PA3390, both ABM domains, were shown to bind heme directly and were found adjacent to or within operons that detoxify glyoxal (Fig. 8), a metabolite by-product of glycolysis and other pathways that can be damaging to cells, both prokaryotic and eukaryotic (109, 125, 126). These two novel ABM domains were unable to degrade heme, as most known heme-binding ABM domains have been shown to do in other pathogens; therefore, their precise functional roles remain to be determined. One possible connection could be that cytochromes (127) and hemoglobin (128) can be modified by glyoxal or methylglyoxal, resulting in conformational changes and an inability to accommodate heme. Although the literature in this area is sparse, one study has suggested a close association between heme and glyoxal/methylglyoxal metabolism (129).

We have established Met-Seq as a powerful tool in identifying novel regulatory networks associated with a cellular metabolite (heme). In doing so, we first describe the building of a heme biosensor and then use the biosensor in conjunction with Met-Seq to reveal many potentially novel genes and pathways related to intracellular heme levels in a major pathogen. Our data presented here are intended to provide a foundation for the use of Met-Seq in identifying genes that directly affect cellular metabolite levels in any biological system where a biosensor is available. For future Met-Seq studies, improvements can certainly be made using more saturating and unbiased library technologies such as barcoding (130, 131). A recent publication has utilized barcoding and an enzyme biosensor, similar to what we describe here, to screen for genes in yeast influencing l-3,4-dihydroxyphenylalanine (l-DOPA) production (23). Here, we have expanded this technology to prokaryotes and, specifically, applications to bacterial pathogens. It is therefore exciting to envision the possibilities Met-Seq could theoretically be extrapolated to for use with any genetically tractable prokaryotic or eukaryotic system to provide data that directly link a given gene product, under a given environmental condition, with metabolic flux. We envision that Met-Seq will enable more rapid discoveries of global metabolic connections, thereby expediting scientific discoveries.

## MATERIALS AND METHODS

### Bacterial growth conditions.

For standard strain maintenance, P. aeruginosa and Escherichia coli liquid cultures were grown in lysogeny broth (LB; Invitrogen, Carlsbad, CA). For growth on solid media, LB solidified with 1.5% agar (No. 1; Oxoid, Hampshire, UK) was used for E. coli, and both LB agar and *Pseudomonas* isolation agar (PIA; Sigma-Aldrich, St. Louis, MO) supplemented with 20 ml/liter glycerol were used for P. aeruginosa. Where LB containing sucrose was required, 5% (wt/vol) sucrose was added after autoclaving. M9 minimal salts was prepared according to the manufacturer’s instructions (Anachem, Leicester, UK). To deplete iron, M9 medium was treated (after autoclaving but prior to the addition of supplements) with 2% (wt/vol) Chelex-100 sodium (Sigma-Aldrich, St. Louis, MO) overnight at 4°C with stirring. Chelex beads were removed by filter sterilization with a 0.2-μm filter. Chelex-treated M9 medium was then supplemented with 0.2% glycerol and 2 mM MgSO_4_ for P. aeruginosa growth (this is referred to as “minimal medium” throughout this study). M-56 medium was made as described in reference 132. Super optimal broth with catabolite repression (SOC) contained 2% (wt/vol) tryptone, 0.5% (wt/vol) yeast extract, 10 mM NaCl, 2.5 mM KCl, 10 mM MgCl_2_, and 20 mM glucose. Vogel-Bonner medium E (VBM) was prepared as per the recipe described in reference 133 at 50× stock solution. Hemin stock solutions were prepared as described in reference 134. For P. aeruginosa, the following antibiotics and concentrations were used: carbenicillin (Cb), 250 μg/ml; gentamicin (Gm), 30 to 75 μg/ml; streptomycin (Sm), 2,000 μg/ml. For E. coli, 50 μg/ml Cb, 50 μg/ml kanamycin (Kan), 30 μg/ml Gm, and 50 μg/ml Sm were used.

### P. aeruginosa growth and fluorescence assays.

P. aeruginosa growth and fluorescence assays were performed in either 250-ml Erlenmeyer flasks in a shaking incubator or, alternatively, in microtiter plates in a microtiter plate reader, which could also act as a temperature and gas-regulated air shaker. For plate reader assays, an Infinite PRO M200 with an extended red spectrum photomultiplier was used (Tecan, Männedorf, Switzerland), with the exception of experiments shown in Fig. 1E and F, and Fig. S1F and G, where a BioTek Synergy H1 multimode plate reader (also with extended red spectrum photomultiplier) was used (BioTek, Winooski, VT).

Optical density at 600 nm (OD_600_) was measured using a Biomate 3 spectrophotometer (Fisher Scientific, Waltham, MA), and PBF fluorescence was either measured using a microtiter plate reader or assessed by flow cytometry (see below). For assays performed with iron-depleted M9 minimal medium (Fig. 1G and H and 2B to D; see also Fig. S1E, Fig. S3A to C, E, F and Fig. S7 in the supplemental material), overnight cultures of P. aeruginosa cells were resuspended to an OD of 0.2 and starved of iron via growth in minimal medium in the absence of an iron source for 4 h at 37°C with 230 rpm shaking. After 4 h, hemin was added to a final concentration of 5 μM and PBF plus HO expression was induced with the addition of 0.2% (wt/vol) arabinose. For assays performed with LB/M-56, expression of the PBF plus HO synthetic operon from the pIFPHO plasmid was induced by the addition of 0.2% arabinose (wt/vol) at time zero (after resuspension). Optical density and PBF fluorescence together (i.e., fluorescence density) was measured every 1 to 2 h as described above. For assays performed in microtiter plates, overnight cultures were pelleted at 1,700 × *g* for 10 min, followed by a washing step using fresh growth medium. Cells were then resuspended in the above-indicated medium with or without the relevant supplements (e.g., ALA or hemin) and arabinose at 0.2% (wt/vol) to an OD_600_ of approximately 0.1 (for growth in LB) or 0.2 (for growth in minimal medium). Cells were then grown in 96-well black μClear microtiter plates (Greiner Bio-One, Kremsmünster, Austria) in either an InfinitePRO microtiter plate reader (Tecan) or a BioTek Synergy H1 multimode plate reader (BioTek) in 100- to 200-μl volumes in triplicates. Plates were incubated at 37°C and aerated by 452 rpm linear shaking throughout the assay.

10.1128/mSystems.00933-20.7 FIG S7 Construction and verification of pIFPHO_AR. (A) pIFPHO was modified to express an ampicillin (Amp) resistance selectable marker (*bla*; from pUCP19) in addition to the Gm resistance marker (*aacC1*). *Bla* along with its promoter region was inserted at the PstI site, downstream of the IFP-HO construct, to yield the plasmid pIFPHO_AR. Growth (B) and fluorescence density growth curves (C) of WT MPAO1, *ΔphuR*, and *ΔphuUV* strains containing pIFPHO_AR. Means ± SDs from three technical repeats are shown. The curves are representative of three biological repeats. (D) Fluorescence density of strains in panel C shown as a percentage of WT (set to 100%) after 11 h pf growth. The means ± SDs from three biological repeats are shown. *, *P* < 0.05 as determined by a one-sample *t* test. (E) Verification of *holo*-phytochrome (PBFs) expression via zinc blot. Proteins from whole-cell lysate were separated by SDS-PAGE and incubated with zinc acetate buffer, and the presence of IFP1.4 was visualized by UV-induced fluorescence (top). The corresponding Coomassie brilliant blue-stained gel is shown at the bottom. Gels are representative of three biological repeats. +, cells harboring the biosensor plasmid. Download FIG S7, EPS file, 1.5 MB. Copyright © 2021 Glanville et al. 2021 Glanville et al. This content is distributed under the terms of the Creative Commons Attribution 4.0 International license.

### Construction of pIFP, pIFPHO, and pIFPHO_AR.

pIFP and pIFPHO plasmids were constructed using the pSB109 E. coli-P. aeruginosa shuttle vector plasmid as a backbone (135) (Fig. 1A; Table S3). Prior to constructing pIFP and pIFPHO, we poisoned the latter NcoI site by introducing a point mutation using a variation on the QuikChange method (Stratagene, La Jolla, CA) (Table S3). A synthetic operon construct was synthesized by GenScript (Piscataway, NJ) consisting of the gene encoding the IFP1.4 fluorophore (16) and a 3′ heme oxygenase (HemO) from the cyanobacteria *Synechocystis* sp. strain PCC 6803 preceded by a strong ribosome binding site (RBS). The IFP and IFP1.4-HO constructs were amplified by PCR and ligated into pSB109 digested with NcoI and NdeI (New England Biolabs, Ipswich, MA) using a restrictionless cloning method (136). Two separate PCRs were run to generate NcoI and NdeI cohesive ends using primer pairs IFP F1/IFP R2 and IFP F2/IFP R1 for IFP and IFP F1/IFPHO R2 and IFP F2/IFPHO R1 for IFPHO (Table S3). The reverse primers include a C-terminal FLAG epitope tag to facilitate detection of IFP1.4 when expressed from pIFP or the HO in the case of pIFPHO.

10.1128/mSystems.00933-20.10 TABLE S3 Strains, plasmids, and oligonucleotides. Gm^r^, gentamicin resistant; Amp^r^, ampicillin resistant; Amp^s^, ampicillin sensitive. Download Table S3, PDF file, 0.2 MB. Copyright © 2021 Glanville et al. 2021 Glanville et al. This content is distributed under the terms of the Creative Commons Attribution 4.0 International license.

To construct pIFPHO_AR, the parent pIFPHO plasmid was digested with PstI (New England Biolabs), which cuts at a single site within the multiple cloning site of pIFPHO downstream of the *IFP-HO* operon. The *bla* (ampicillin) resistance gene and its promoter were amplified by PCR from pUCP19 using primers pUCP19_bla_F/pUCP19_bla_R (Table S3), which introduce PstI restriction sites to the ends of the DNA product. Following PstI digestion (New England Biolabs), the resulting DNA product was ligated into the PstI site of pSB109, yielding pIFPHO_AR, which expresses both gentamicin (*accC1*) and ampicillin (*bla*) resistance markers (Fig. S7A). Successful insertion of *bla* into pIFPHO was confirmed by growth of pIFPHO_AR on LB agar containing 50 μg/ml carbenicillin followed by DNA sequencing. pIFPHO_AR was then transformed into WT MPAO1, Δ*phuUV*, and Δ*phuR* strains and tested for growth and fluorescence density signal over time. It was then verified that strains harboring pIFPHO_AR grew as expected (Fig. S7B). In addition, the expected fluorescence density differences were observed that paralleled that of the parent pIFPHO biosensor plasmid (Fig. S7C, D), indicating the slightly modified pIFPHO_AR vector could be used for Met-Seq library construction.

### Generation of P. aeruginosa deletion mutants.

Clean deletions of genes of interest in P. aeruginosa were constructed essentially as described in reference 137. DNA primers were designed to amplify the 500-bp region 5′ to the gene of interest containing the first three codons of the gene to be deleted (designated primer 1 and 2) (Table S3) and a 500 bp region 3′ to the gene of interest containing the last three codons of the gene of interest (designated primers 3 and 4) (Table S3). Primers 2 and 3 were designed such that they also contained regions complementary to each other, allowing overlap extension PCR to be performed to join the 500 bp upstream and downstream regions to create a “mutator fragment.” All primers were designed such that no part of the coding region or ribosome binding sites of neighboring genes were disrupted. PCRs were carried out using either Phusion high-fidelity DNA polymerase (New England Biolabs, Ipswich, MA) or Platinum Pfx DNA polymerase (Life Technologies).

The resultant mutator fragments were then ligated into the pCR2.1 plasmid (Thermo Fisher Scientific) using T4 DNA ligase (New England Biolabs) and then subcloned into pKNG101 (Table S3) using two of the following sites: ApaI, SpeI, and BamHI. Successful ligation of the mutator fragments into pKNG101 was confirmed by restriction digest. To generate the HasR, PA0028, PA0149, and PA0527 (Dnr) mutator products, cassettes were first synthesized by GenScript in the vector pUC57 (for HasR) or as linear DNA fragments (for PA0028, PA0149, and PA0527), which were subsequently ligated into pUC19. The cassettes were then excised by restriction enzyme digestion and ligated into pKNG101. For all mutants, pKNG101 containing the relevant mutator fragment was transformed into E. coli CC118 λ*pir* for mobilization into MPAO1 using E. coli 1047 cells containing pRK2013 (138). MPAO1 transformants were confirmed by colony PCR using gene specific primers 1 plus 4 and 5 plus 6 (Table S3) and sequencing (GATC Biotech, Cologne, Germany). Biosensors were introduced into WT MPAO1 by electroporation as previously described (139), and expression of *holo* PBFs (i.e., uniform incorporation of the biliverdin chromophore) was confirmed by zinc blot and Coomassie brilliant blue staining as per reference 140 (Fig. S7E).

### Transposon mutant library construction.

For pIFPHO_AR compatibility, we needed to remove the *bla* gene from pBT20, the vector used to generate the transposon library. pBT20 harbors both gentamicin (*aacC1*) and ampicillin (*bla*) selectable markers and was thus digested with SpeI and PvuI restriction enzymes to excise the Bla coding sequence. This product was then treated with T4 DNA polymerase (NEB) to generate blunt ends and finally religated with T4 DNA ligase (Promega) to generate pBT20_AS. Loss of the *bla* gene was confirmed by patching the resulting transformants on LB agar containing 50 μg/ml Cb. The MPAO1 transposon library was then constructed with pBT20_AS (Table S3) as previously described (141).

### Fluorescence analysis of individual clones.

For plate reader detection, PBF fluorescence was measured either in an InfinitePRO microplate reader (Tecan, Männedorf, Switzerland), or a BioTek Synergy H1 multimode plate reader (BioTek, Winooski, VT). Bacterial cells were measured in 100- to 200-μl volumes in a 96-well Black μClear microtiter plate (Greiner Bio-One, Kremsmünster, Austria). PBFs were excited at 675 (bandwidth 9) nm and fluorescence detected at 725 (bandwidth 20) nm. Data were processed via the Tecan Magellen or Biotek Gen5 software packages and exported to Microsoft Excel for analysis. For flow cytometry, P. aeruginosa cells were fixed in 4% paraformaldehyde (PFA) for 10 min at room temperature and stored in phosphate-buffered saline (PBS). Cells were then analyzed on an LSRFortessa (BD Biosciences, San Jose, CA) cell analyzer using a gate based on side scatter (SSC-H) at a voltage of 200. PBFs were excited with a 640 nm laser, and fluorescence was collected using a 730/45 nm band pass filter. Data were analyzed using FlowJo 8.6.3 or FlowJo 10.0 software (FlowJo LLC, Ashland, OR).

### Fluorescence-activated cell sorting of the PBF-expressing Tn library.

To enrich for PBF fluorescence, glycerol stocks of the previously stored library populations were defrosted on ice and used to inoculate minimal medium to an OD_600_ of approximately 0.2. Cells were starved for 4 h without an iron source. After this, cells were supplemented with 5 μM heme and 0.2% (wt/vol) arabinose and grown as described above. After 14 h growth, cells were pelleted at 4,000 × *g* for 10 min, followed by resuspension in sterile PBS. Cell reporter fluorescence was measured on a FACSAria III (BD Biosciences), equipped with a 633 nm laser and Alexa Flour 700 filters (730/45 nm). Library cells that exhibited diminished (dim), similar (mid), or enhanced (bright) fluorescence compared to that of the WT were determined by eye and gated into separate populations. At least 2 × 10^5^ events were collected in each gate. Sorted cells were recovered by plating on LB agar containing 250 μg/ml Cb and incubating overnight at 37°C. Colonies were subsequently scraped from the agar plates and stored in LB containing 15% glycerol at −80°C to be either used for the next round of enrichment or sequenced.

### Transposon sequencing.

Genomic DNA (gDNA) for transposon sequencing was isolated from FACS populations (the initial pre-FACS library and enrichments E1 to -3) by phenol-chloroform extraction. To process the gDNA samples, RNA contamination was first removed by RNase A treatment (Fisher Scientific, Waltham, MA), and then the samples were further prepared for sequencing essentially as previously described (142). Sequencing reads containing the transposon were collected using FASTX Barcode Splitter (FASTX-Toolkit) and then mapped to the P. aeruginosa PAO1 genome using Bowtie version 0.12.8 with default parameters, except that only one read was reported (at random) for reads with more than one reportable alignment.

### Met-Seq data manipulation and statistical analyses.

Overall, we received fewer sequencing reads than expected with our MiSeq runs of all samples. Based on this initial observation, we then needed to test if the low number of unique Tn insertions could be problematic for accurate statistical analyses. To test this, we randomly sampled the mapped reads for the library to various depths and then analyzed these in a one-sample analysis using TSAS (41). This was conducted to identify the number of unique Tn insertions at each tested depth. When comparing the reads versus insertions, we observed that having more sequencing reads in our MiSeq run would not have uncovered significantly more unique insertions, confirming that our sequencing coverage was sufficient for a library of our complexity (Fig. 2E and F). Thus, changing the minimum read threshold did not have a significant impact on the final E3 output list. Even so, we still applied a stringent minimum threshold of at least 10 reads to the finalized gene output file (Table S1A to C), as per reference 41. Seven hundred thirteen insertion sites in total were identified in the E3 population, with the number of reads at single Tn insertion site ranging from 1 to 341,474. The average number of reads per insert in the E3 population was 1,062, and the median was 65 (with the mode at 1 read).

Statistics displayed in Table S1 were computed by comparing the control (presorted MPAO1 Tn library) to the treatment (E3 sorted population only). Statistical categories included average unique hits (Tn insertions), average raw reads, ratio of insertions (treatment/control), log_2_ fold change in insertions, log_2_ fold change in reads, and *P* values. *P* values from a binomial distribution assessing the likelihood of genes whose disruption by transposon insertions may have resulted in an overall improvement of fitness were calculated by comparing the proportions of insertions in the E3 population versus that in the library control. These *P* values where then adjusted (Adj. *P* value column) using the Benjamini-Hochberg (BH) method as described in reference 41. Weighted outputs were not used, as these data would bias larger genes that would have a statistically greater chance of having more Tn insertions. The data were sorted on log_2_ fold change in overall reads per gene before being displayed in Table S1. Data were exported as WIG files to upload into MochiView (143) for analyses and illustrated representations.

### Computational analyses of Met-Seq data.

Protein-protein interaction data for P. aeruginosa was downloaded from STRING DB v11.0 (https://string-db.org/) (44). STRING protein identifiers were mapped to NCBI gene identifiers (IDs) and then to locus tags using P. aeruginosa genomes GCA_000006765.1_ASM676v1 and GCA_000743405.1_PAG. The top 20% of all STRING-predicted P. aeruginosa protein-protein interactions were retained. This corresponded to a combined score cutoff of 317 and a total of 356,372 interactions. All unique interactions between the proteins encoded by the 188 enriched ORFs (Table S1A) were then extracted from this list, resulting in a total of 244 unique protein-protein interactions. The resulting network map was plotted in Cytoscape 3.7.2 (144). The enriched ORFs, as well as gene clusters in the STRING network, were tested for functional enrichment via overrepresentation analysis using the hypergeometric distribution. P. aeruginosa pathways and GO terms were obtained from the *Pseudomonas* genome database (https://www.pseudomonas.com) (145). Additional functional enrichment analysis was conducted with the DAVID bioinformatics database using genes in Table S1A (47).

### Determination of intracellular heme concentrations.

Intracellular heme concentrations were determined by either a pyridine hemochrome assay (57) or a fluorometric assay (30). For the pyridine hemochrome assay, 12 ml of LB in a 125-ml Erlenmeyer flask was inoculated with a single colony of P. aeruginosa from an LB plate and shaken overnight at 37°C. Forty milliliters of culture at an OD_600_ of 0.2 was then transferred by successive centrifugations into 2-ml microcentrifuge tubes. Supernatants were removed, and pellets were then transferred into 50-ml conical tubes containing 4 ml of M9, washed twice more in 5 ml of M9, and then finally resuspended in 1 ml of M9 and transferred to 250-ml Erlenmeyer flasks containing 39 ml of fresh M9 medium. Cultures were then shaken at 37°C for 4 h to starve cells of iron. After 4 h, hemin was then added to cultures to a final concentration of 5 μM, and the cells were shaken at 37°C for an additional 10 h. After 10 h, flasks were immediately placed on ice and the OD_600_ was read. Cultures were then transferred to 50-ml conical tubes (Corning), pelleted, washed twice in PBS, and finally resuspended in fresh PBS to an OD_600_ of 10. Samples were then pelleted in 1-ml tubes and stored at −80°C at a stopping point.

To read the absorbance, samples were thawed and resuspended in 840 μl of B-PER reagent (Thermo Scientific) with 1 mg/ml lysozyme (Millipore Sigma), 4 μg/ml DNase I (Roche Diagnostics), and 1.2 μg/ml RNase A (Sigma-Aldrich, St. Louis, MO). Samples were then incubated at room temperature with constant mixing for 30 min. Two hundred microliters of pyridine (Fisher Chemicals) and 100 μl of 1 M NaOH were then added to the samples on ice, followed by addition of 10 μl of 1 M potassium ferricyanide (Acros Organics) in order to oxidize the sample. Samples were then transferred to 1.4-ml quartz cuvettes, and absorbance spectra were obtained on a Shimadzu UV-1650PC spectrophotometer, which was blanked to a cuvette with assay reagents only. Samples were then scanned from 400 to 700 nm at wavelength intervals of 0.5 nm. Two to five milligrams of sodium dithionite (Acros Organics) was then added and mixed by pipetting to reduce the sample. Absorbance spectra were then similarly obtained for the reduced sample. Heme calculations were then calculated as per reference 57.

To determine intracellular heme by fluorescence, we used a protocol which originated from reference 30 and was performed essentially as described in ref 146. P. aeruginosa was grown in M9 minimal medium containing 5 μM hemin as the sole iron source as described above. After 14 h of growth, 20 ml of culture was pelleted and washed twice in the same volume of PBS. After the second wash, the pellet was resuspended in 21 ml PBS, the OD_600_ was measured, and then 4 × 5-ml pellets were saved for measurement of total cellular heme. Pellets were resuspended in 500 μl of 20 mM oxalic acid (Sigma-Aldrich) and incubated overnight (16 to 24 h) at 4°C in amber 1.5-ml tubes. Next, 500 μl of 2 M oxalic acid was added and mixed. Five hundred microliters of each sample was then put aside in another amber 1.5-ml tube and kept in an opaque box at room temperature. The other 500 μl was boiled at 98°C for 30 min. Both boiled and nonboiled samples were then clarified by centrifugation at full speed. Two hundred microliters of supernatant was taken, and fluorescence (excitation 400 nm, emission 620 nm) was measured in a 96-well black μClear microtiter plate (Greiner Bio-One, Kremsmünster, Austria) using a BioTek Synergy H1 multimode plate reader (BioTek, Winooski, VT). The nonboiled sample acted as a blank for the boiled sample. Fluorescence measurements were normalized using the culture density (OD_600_). Independent biological replicates were used and at least three technical replicates were performed for each biological replicate.

### Construction of pET15DG1.

To construct pET15DG1, pET-15b (Novagen, EMD Millipore) was modified. The DNA sequence between the NcoI and NdeI sites of pET-15b encodes a thrombin cleavage site. To change this site to a tobacco etch virus (TEV) protease cleavage site, complementary oligonucleotides that encode the TEV protease site and that, when annealed, generate NcoI and NdeI sticky ends, were synthesized (pET15b TEV F/pET15b TEV R) (Table S3). The two oligonucleotides were annealed by mixing at an equal molar ratio, and the resulting product was phosphorylated using T4 polynucleotide kinase (Promega, Madison, WI) in a reaction mixture supplemented with 1 mM ATP at 37°C for 4 h. The phosphorylated products were then cleaned again using QIAquick PCR purification columns and then ligated in pET-15b that had been digested with NcoI-HF and NdeI (NEB, Ipswich, MA) using T4 DNA ligase and LigaFast rapid DNA ligation buffer (Promega) for 5 min at room temperature. Ligation reaction mixtures were then transformed into E. coli DH5α chemically competent cells and plated on LB agar containing 50 μg/ml carbenicillin. Clones were verified by DNA sequencing.

### Protein expression strain construction.

PA0709 and PA3390 DNA from P. aeruginosa strain MPAO1 was amplified and ligated into the NdeI and BlpI sites of pET15DG1 by a restrictionless cloning method (136). Two separate reactions were run to generate cohesive ends using primer pairs PA0709-F1/PA0709-R2 and PA0709-F2/PA0709-R1, or PA3390-F1/PA3390-R2 and PA3390-F2/PA3390-R1 (Table S3). The two reactions were then combined and purified using QIAquick PCR purification columns (Qiagen, Valencia, CA) and eluted in 40 μl of double-distilled water (ddH_2_O). Using a thermocycler, the combined insert DNA was melted at 98°C and slowly reannealed to generate the NdeI and BlpI cohesive ends. Next, the PA0709/PA3390 products were phosphorylated using polynucleotide kinase (Promega, Madison, WI) in a reaction mixture supplemented with 1 mM ATP at 37°C for 4 h. The phosphorylated products were then cleaned again using QIAquick PCR purification columns and then ligated into pET15DG1 digested with NdeI and BlpI using T4 DNA ligase and LigaFast rapid DNA ligation buffer (Promega) for 5 min at room temperature. Ligation reactions were then transformed into E. coli DH5α. chemically competent cells and plated on LB agar containing 50 μg/ml carbenicillin. Clones were verified by DNA sequencing. To generate the final protein expression strains, the resultant plasmids were transformed into E. coli T7 Express *lysY^i/q^* cells (New England Biolabs Inc., Ipswich, MA).

### Protein expression and purification.

Expression and purification of PA0709 and PA3390 were carried out as described in reference 147. His_6_-tagged PA0709 and PA3390 proteins were expressed in E. coli T7 Express *lysY^i/q^* cells (New England BioLabs Inc., Ipswich, MA) carrying pET15DG1-PA0709 or pET15DG1-PA3390. Cultures were grown at 37°C with shaking at 230 rpm in LB medium supplemented with 50 μg/ml carbenicillin. When cultures reached an OD_600_ of ∼0.8, protein expression was induced by the addition of 1 mM isopropyl-β-d-thiogalactopyranoside (IPTG). The temperature was then reduced to 18°C, and the cultures were grown overnight with shaking at 150 rpm. The following morning, cells were harvested by centrifugation and resuspended in 25 ml of lysis/wash buffer (50 mM Tris-HCl [pH 8.0], 50 mM imidazole, 1 mM β-mercaptoethanol [BME]) supplemented with 1 mM phenylmethylsulfonyl fluoride (PMSF) and one cOmplete mini protease inhibitor tablet (Sigma-Aldrich, St. Louis, MO). Cells were lysed by sonication using a Branson Sonifier S-450 cell disruptor (Branson Ultrasonics Corp., Danbury, CT) for a total of 2 min of sonication at 75% amplitude with 15-s pulses, separated by 5-min rest periods on ice. The lysate was then clarified by centrifugation at 27,000 × *g* for 45 min in a Sorvall SS-34 rotor (Thermo Fisher scientific). To isolate the recombinant His_6_-tagged PA0709 protein, the clarified lysate was applied to 1 ml of Ni-nitrilotriacetic acid (NTA)-agarose (Qiagen, Valencia, CA) by gravity flow. To remove contaminating proteins, the column was washed with 50 ml lysis/wash buffer. Recombinant proteins were eluted in 20 ml of elution buffer (150 mM Tris-HCl [pH 8.0], 300 mM imidazole, 1 mM BME) in 1-ml fractions. The purity of the fractions was assessed by SDS-PAGE and Coomassie staining before pooling. Proteins were then dialyzed against 4 liters of storage buffer (150 mM Tris-HCl [pH 8.0], 1 mM BME, 5% glycerol) overnight before storage at −80°C. The resulting preparations were >95% pure.

### Heme binding experiments.

Heme binding experiments were performed as described in reference 107 with some modifications. Aliquots of hemin were added to 10 μM apo-PA0709 or apo-PA3390 to a final concentration of 2 to 40 μM. After a 5-min incubation, 3 × 200 μl of each binding reaction was transferred to a 96-well plate, and a spectral scan of the absorbance between 300 nm and 800 nm with 2-nm increments was performed in a BioTek Synergy H1 multimode plate reader (BioTek, Winooski, VT).

### Single-turnover heme degradation experiments.

Heme degradation experiments were carried out as described in reference 107 with some modifications. Briefly, recombinant *apo*-PA0709 or *apo*-PA3390 proteins were reconstituted with hemin to a molar ratio of 1:1 by dropwise addition of 400 μM hemin in 150 mM Tris-HCl (pH 8.0) into solutions of either *apo*-PA0709 or *apo*-PA3390 in the same buffer. Samples were incubated on ice for 1 h before desalting on a 10 ml Zeba 7-kDa molecular weight cutoff (MWCO) desalting column (Thermo Fisher Scientific). Protein concentration was then read by Bradford assay. Catalase (MP Biomedicals LLC, Solon, OH) was then added to a 0.5:1 ratio (catalase to protein) to prevent nonenzymatic degradation. The samples were then diluted in 150 mM Tris-HCl (pH 8.0) to a final concentration of 10 μM with either PA0709 or PA3390 protein. The electron donor, l-ascorbic acid (Fisher Scientific), was then added to a final concentration of 10 mM. Two-hundred-microliter reactions were performed in 96-well plates. Degradation was monitored by taking a spectral scan of the absorbance between 300 nm and 700 nm with 2-nm increments in a BioTek Synergy H1 multimode plate reader (BioTek, Winooski, VT) every 5 min for 1 h. Reactions were performed in triplicates.

### Western blot analysis.

For Western blot analysis, protein extracts were separated by SDS-PAGE and then transferred to polyvinylidene difluoride (PVDF) membranes (EMD Millipore). To probe for FLAG-tagged proteins, an M2 mouse anti-FLAG monoclonal antibody (Sigma-Aldrich, St. Louis, MO) was used at a dilution of 1:10,000 with overnight incubation at 4°C. Proteins of interest were detected using horseradish peroxidase (HRP)-conjugated goat anti-mouse IgG used at a dilution of 1:5,000 (Jackson ImmunoResearch, West Grove, PA) and visualized with Pierce ECL Western blotting substrate (Thermo Scientific). Blots were imaged using a ProteinSimple FluorChem E imager (ProteinSimple).

### Data availability.

Transposon-generated library and E1, E2, and E3 enrichments have been deposited in the NCBI Sequence Read Archive (SRA) (https://www.ncbi.nlm.nih.gov/sra) as BioProject ID PRJNA685600. Tn-seq analysis software (TSAS) used in this study is in reference 41. TSAS software code can be accessed at GitHub with the following URL: https://github.com/srimam/TSAS.
