# Supplementary material for: A High-Throughput Method for Identifying Novel Genes That Influence Metabolic Pathways Reveals New Iron and Heme Regulation in Pseudomonas aeruginosa
Source: mSystems. 2021 Feb 2;6(1):e00933-20. doi: 10.1128/mSystems.00933-20 (PMC7857532; doi:10.1128/mSystems.00933-20)
Supplement: TABLE S1 [file mSystems.00933-20-st001.pdf]

Table S1A. Met-Seq Enriched Open Reading Frames*

Gene ID**	Name	Category ***	Annotation	Ave Unique hits (treatment)	Ave. Unique hits (control)	Ave. Raw Reads (treatment)	Ave. Raw Read (control)	Ratio_ Insertions (Treatment/ control)	Log2-fold Change (Insertions)	Ratio_ reads (Treatment /control)	Log2-fold Change (reads)	pvalue (proportions_ reads)	Adj. pvalue (proportions_ reads)
PA0709		3	ABM domain protein	1.00	0.00	136.00	0.00	9.72	3.28	41.32	5.37	1.29E-10	2.14E-09
PA0747		3	Probable aldehyde dehydrogenase	1.00	0.00	134.00	0.00	9.72	3.28	41.32	5.37	1.29E-10	2.14E-09
PA0959		10	HIT domain protein	1.00	0.00	192.00	0.00	9.72	3.28	41.32	5.37	1.29E-10	2.14E-09
PA3751	PurT	3	Phosphoribosylglycinamide formyltransferase 2 (purine biosynthesis)	1.00	0.00	162.00	0.00	9.72	3.28	41.32	5.37	1.29E-10	2.14E-09
PA3798		3	PLP-dependent aminotransferase or kynurenine aminotransferase (putative)	1.00	0.00	119.00	0.00	9.72	3.28	41.32	5.37	1.29E-10	2.14E-09
PA5457	WbdD	3	Common polysaccaride antigen (CPA) biosynthesis (O-antigen synthesis methyltransferase)	1.00	0.00	621.00	0.00	9.72	3.28	41.32	5.37	1.29E-10	2.14E-09
PA1963		16	Hydrophobic peptide required for survival in oxygen	1.00	0.00	23.00	0.00	9.72	3.28	14.62	3.87	1.31E-04	1.46E-04
PA1108		9	Tet resistance protein (transporter)	1.00	0.00	22.00	0.00	9.72	3.28	13.98	3.81	1.84E-04	2.04E-04
PA0527	Dnr	13	Transcriptional regulator Dnr	1.00	0.00	10.00	0.00	9.72	3.28	6.36	2.67	1.17E-02	1.24E-02
PA0028		16	Phosphatidylethanolamine (PE) lipid taxis system (3' to HemF operon)	1.00	1.00	147.00	91.00	1.00	0.00	4.13	2.05	5.74E-06	2.86E-05
PA0099		8	Vrgb1-dependent nuclease toxin secreted by T6SS	1.00	1.00	272.00	95.00	1.00	0.00	4.13	2.05	5.74E-06	2.86E-05
PA0149		13	Sigma-70 factor, ECF subfamily; Regulates iron/metal uptake	1.00	1.00	131.00	165.00	1.00	0.00	4.13	2.05	5.74E-06	2.86E-05
PA0182	FabG-like	11	FabG homolog, function unknown	1.00	1.00	141.00	28.00	1.00	0.00	4.13	2.05	5.74E-06	2.86E-05
PA0249		7	Predicted GNAT acetyltransferase	1.00	1.00	551.00	328.00	1.00	0.00	4.13	2.05	5.74E-06	2.86E-05
PA0277		9	HtpX Zn-dependent protease chaperone	1.00	1.00	166.00	95.00	1.00	0.00	4.13	2.05	5.74E-06	2.86E-05
PA0278	TauE/ SafE	9	Sulfite exporter TauE/SafE	1.00	1.00	378.00	363.00	1.00	0.00	4.13	2.05	5.74E-06	2.86E-05
PA0281	CysW	9	Sulfate transporter CysW	1.00	1.00	106.00	179.00	1.00	0.00	4.13	2.05	5.74E-06	2.86E-05
PA0319		16	Part of the *car* calcium responsive operon	1.00	1.00	192.00	74.00	1.00	0.00	4.13	2.05	5.74E-06	2.86E-05
PA0491		13	Predicted LysR type transcriptional regulator of unknown function	1.00	1.00	1581.00	6208.00	1.00	0.00	4.13	2.05	5.74E-06	2.86E-05
PA0765	MucC	13	Positive regulator for alginate biosynthesis	1.00	1.00	1338.00	170.00	1.00	0.00	4.13	2.05	5.74E-06	2.86E-05
PA0887	AcsA	3	Acetyl-CoA synthetase	1.00	1.00	126.00	132.00	1.00	0.00	4.13	2.05	5.74E-06	2.86E-05
PA0920	MprF	16	Bacterial defensin resistance protein (virulence factor); In operon with VirJ	1.00	1.00	113.00	10.00	1.00	0.00	4.13	2.05	5.74E-06	2.86E-05
PA1001	PhnA	1	Anthranilate synthase component I	2.00	2.00	351.00	164.00	4.86	2.28	4.13	2.05	1.41E-10	2.16E-09
PA1141		13	LysR-type transcriptional regulator, similar fold to OxyR	1.00	1.00	187.00	188.00	1.00	0.00	4.13	2.05	5.74E-06	2.86E-05
PA1169		8	Arachidonate 15-lipoxygenase	1.00	1.00	175.00	83.00	1.00	0.00	4.13	2.05	5.74E-06	2.86E-05
PA1179	PhoP	13	Two-component response regulator PhoP	1.00	1.00	91.00	33.00	1.00	0.00	4.13	2.05	5.74E-06	2.86E-05
PA1192	TilS/ MesJ	3	Probable lysidine synthase TilS/MesJ	1.00	1.00	679.00	359.00	1.00	0.00	4.13	2.05	5.74E-06	2.86E-05
PA1218	PhyH	3	Phytanoyl-CoA dioxygenase; binding of 2-oxoglutarate to the iron(II) containing enzyme	1.00	1.00	99.00	29.00	1.00	0.00	4.13	2.05	5.74E-06	2.86E-05
PA1247	AprE	9	Alkaline protease secretion protein AprE	1.00	1.00	137.00	44.00	1.00	0.00	4.13	2.05	5.74E-06	2.86E-05
PA1455	FliA	2	Flagellar biosynthesis sigma factor	1.00	1.00	1347.00	407.00	1.00	0.00	4.13	2.05	5.74E-06	2.86E-05
PA1510	PGAP1-like	8	T6SS phospholipase effector - targets bacterial cells and induced ER/autophagy	1.00	1.00	172.00	40.00	1.00	0.00	4.13	2.05	5.74E-06	2.86E-05
PA1538	CzcO	16	Associated with the cation diffusion facilitator CzcD	1.00	1.00	173.00	30.00	1.00	0.00	4.13	2.05	5.74E-06	2.86E-05
PA1708	PopB	8	Translocator protein PopB, Type III Secretion System protein	1.00	1.00	1453.00	154.00	1.00	0.00	4.13	2.05	5.74E-06	2.86E-05
PA1758	PabB	3	Para-aminobenzoate synthase (chorismate metabolism)	1.00	1.00	108.00	65.00	1.00	0.00	4.13	2.05	5.74E-06	2.86E-05
PA1941		16	Predicted cytochrome	3.00	3.00	1244.00	194.00	4.86	2.28	4.13	2.05	4.00E-15	1.65E-13
PA1991	ErcA	3	Iron-containing alcohol dehydrogenase	1.00	1.00	275.00	48.00	1.00	0.00	4.13	2.05	5.74E-06	2.86E-05
PA2349		9	D-methionine transport system operon	1.00	1.00	77.00	59.00	1.00	0.00	4.13	2.05	5.74E-06	2.86E-05
PA2464	FoxB	16	FoxB siderophore operon	1.00	1.00	412.00	529.00	1.00	0.00	4.13	2.05	5.74E-06	2.86E-05
PA2708		16	Unknown but contains a DUF748	1.00	1.00	346.00	407.00	1.00	0.00	4.13	2.05	5.74E-06	2.86E-05
PA2813	GST	10	Glutathione S-transferase	1.00	1.00	1011.00	737.00	1.00	0.00	4.13	2.05	5.74E-06	2.86E-05
PA2843	DAHP synth	3	Chorismate synthesis (catalyzes first step)	1.00	1.00	195.00	110.00	1.00	0.00	4.13	2.05	5.74E-06	2.86E-05
PA2896	SbrR/ SbrI	2	Sigma and anti-sigma factor that regulate biofilms and motility	1.00	1.00	297.00	64.00	1.00	0.00	4.13	2.05	5.74E-06	2.86E-05
PA2902		16	WG reapeat containing protein	1.00	1.00	248.00	134.00	1.00	0.00	4.13	2.05	5.74E-06	2.86E-05

PA2925	HisM	9	Histidine transporter permease HisM	1.00	1.00	231.00	48.00	1.00	0.00	4.13	2.05	5.74E-06	2.86E-05
PA3061	PelD	13	Cyclic-di-GMP binding, regulation of polysaccharide	2.00	2.00	918.00	434.00	4.86	2.28	4.13	2.05	1.41E-10	2.16E-09
PA3218		16	protein of unknown function	1.00	1.00	400.00	185.00	1.00	0.00	4.13	2.05	5.74E-06	2.86E-05
PA3349	CheW	2	Chemotaxis protein	1.00	1.00	691.00	62.00	1.00	0.00	4.13	2.05	5.74E-06	2.86E-05
PA3390		3	ABM domain protein	1.00	1.00	6406.00	1804.00	1.00	0.00	4.13	2.05	5.74E-06	2.86E-05
PA3517	PurB	3	Adenylosuccinate lyase, purine metabolism	1.00	1.00	555.00	11.00	1.00	0.00	4.13	2.05	5.74E-06	2.86E-05
PA3641		9	Predicted glycine transporter	1.00	1.00	545.00	1402.00	1.00	0.00	4.13	2.05	5.74E-06	2.86E-05
PA3765		16	DUF2239 domain - protein function is unknown	1.00	1.00	93.00	108.00	1.00	0.00	4.13	2.05	5.74E-06	2.86E-05
PA4137	OprD	9	Porin	1.00	1.00	430.00	994.00	1.00	0.00	4.13	2.05	5.74E-06	2.86E-05
PA4187		9	Predicted sugar major facilitator superfamily (MFS) transporter	1.00	1.00	183.00	91.00	1.00	0.00	4.13	2.05	5.74E-06	2.86E-05
PA4348		10	Predicted beta lactamase AmpC (PA4347) and FprA homolog (PA4348)	1.00	1.00	145.00	243.00	1.00	0.00	4.13	2.05	5.74E-06	2.86E-05
PA4352		13	Anaerobic energy stress protein/Anr regulated	1.00	1.00	499.00	32.00	1.00	0.00	4.13	2.05	5.74E-06	2.86E-05
PA4516		16	Hypothetical protein with Sel1-like repeat sequences	1.00	1.00	740.00	52.00	1.00	0.00	4.13	2.05	5.74E-06	2.86E-05
PA4613	KatB	10	Catalase regulated by OxyR	1.00	1.00	229.00	173.00	1.00	0.00	4.13	2.05	5.74E-06	2.86E-05
PA4724	GluQ	7	Glutamyl-Q tRNA(Asp) synthetase	1.00	1.00	67.00	13.00	1.00	0.00	4.13	2.05	5.74E-06	2.86E-05
PA4769	Fur	13	Ferric uptake regulator	1.00	1.00	325.00	601.00	1.00	0.00	4.13	2.05	5.74E-06	2.86E-05
PA4802		3	Predicted amidohydrolase	1.00	1.00	169.00	103.00	1.00	0.00	4.13	2.05	5.74E-06	2.86E-05
PA4804	PotE	15	Putrescine export	1.00	1.00	169.00	76.00	1.00	0.00	4.13	2.05	5.74E-06	2.86E-05
PA4904	VanA	3	Vanillate O-demethylase oxygenase	1.00	1.00	125.00	73.00	1.00	0.00	4.13	2.05	5.74E-06	2.86E-05
PA4905	VanB	3	Vanillate O-demethylase	1.00	1.00	209.00	130.00	1.00	0.00	4.13	2.05	5.74E-06	2.86E-05
PA4908		3	Ornithine cyclodeaminase	1.00	1.00	357.00	138.00	1.00	0.00	4.13	2.05	5.74E-06	2.86E-05
PA4919	PcnB1	3	NAD salvage pathway	1.00	1.00	294.00	259.00	1.00	0.00	4.13	2.05	5.74E-06	2.86E-05
PA5093	HutH	3	Histidine/phenylalanine ammonia- lyase	1.00	1.00	416.00	120.00	1.00	0.00	4.13	2.05	5.74E-06	2.86E-05
PA5113		16	Hypothetical protein (DUF3999)	1.00	1.00	541.00	413.00	1.00	0.00	4.13	2.05	5.74E-06	2.86E-05
PA5307		9	Dicarboxylate transport	1.00	1.00	170.00	217.00	1.00	0.00	4.13	2.05	5.74E-06	2.86E-05
PA5348		13	HU-IHF histone-like DNA binding protein	1.00	1.00	2020.00	3828.00	1.00	0.00	4.13	2.05	5.74E-06	2.86E-05
PA5378	CbcX	16	Glycine/betaine transport	1.00	1.00	471.00	209.00	1.00	0.00	4.13	2.05	5.74E-06	2.86E-05
PA5382		13	Predicted transcriptional regulator	1.00	1.00	815.00	160.00	1.00	0.00	4.13	2.05	5.74E-06	2.86E-05
PA5401		9	ABC-type proline/glycine betaine transport system (periplasmic component)	1.00	1.00	154.00	163.00	1.00	0.00	4.13	2.05	5.74E-06	2.86E-05
PA5434	Mtr	9	Tryptophan permease	1.00	1.00	154.00	317.00	1.00	0.00	4.13	2.05	5.74E-06	2.86E-05
PA5477		16	Unknown function, contains DUF2070 and DUF2914 domains	1.00	1.00	1528.00	241.00	1.00	0.00	4.13	2.05	5.74E-06	2.86E-05
PA5517		16	Hypothetical protein	1.00	1.00	305.00	25.00	1.00	0.00	4.13	2.05	5.74E-06	2.86E-05
PA5542		16	Zn2+-dependent imipenemase	1.00	1.00	115.00	45.00	1.00	0.00	4.13	2.05	5.74E-06	2.86E-05
PA0463	CreB	13	Two-component response regulator CreB	1.00	1.00	62.00	227.00	1.00	0.00	3.94	1.98	1.38E-05	5.46E-05
PA0328		16	Putative peptidase	1.00	1.00	58.00	124.00	1.00	0.00	3.69	1.88	4.37E-05	1.72E-04
PA0652	Vfr	13	Vfr (virulence factor regulator of Type III secretion); c-AMP regulated	1.00	1.00	58.00	71.00	1.00	0.00	3.69	1.88	4.37E-05	1.72E-04
PA0511	NirJ	4	Heme d1 biosynthesis protein	1.00	1.00	56.00	352.00	1.00	0.00	3.56	1.83	7.75E-05	8.60E-05
PA3732		16	Hypothetical protein (DUF2170)	1.00	1.00	56.00	105.00	1.00	0.00	3.56	1.83	7.75E-05	8.60E-05
PA5219	AraJ	9	AraJ (arabinose efflux transporter)	1.00	1.00	52.00	22.00	1.00	0.00	3.31	1.72	2.40E-04	2.65E-04
PA2661		8	Hypothetical protein in operon with Patatin-like phospholipase (PA2660)	1.00	1.00	49.00	114.00	1.00	0.00	3.11	1.64	5.54E-04	6.11E-04
PA2960	PilZ	2	Type 4 fimbrial biogenesis protein	6.00	8.00	2573.00	2197.00	3.65	1.87	3.10	1.63	0.00E+00	0.00E+00
PA1140		3	Glyoxylate-induced protein; PDB code: 1sq4	1.00	1.00	48.00	139.00	1.00	0.00	3.05	1.61	7.30E-04	8.04E-04
PA2755		8	Ecotin; serine protease inhibitor, protects from neutrophil elastase	1.00	1.00	48.00	17.00	1.00	0.00	3.05	1.61	7.30E-04	8.04E-04
PA4362		9	Sugar transport operon	1.00	1.00	47.00	44.00	1.00	0.00	2.99	1.58	9.60E-04	1.05E-03
PA5441		16	Hypothetical protein	1.00	1.00	44.00	28.00	1.00	0.00	2.80	1.48	2.16E-03	2.37E-03
PA2762		16	Cupin family protein, possible ring-cleaving dioxygenase	2.00	3.00	658.00	310.00	3.24	1.70	2.75	1.46	1.79E-07	9.04E-07
PA5042	PilO	2	Type 4 fimbrial biogenesis protein	8.00	12.00	58691.00	10412.00	3.24	1.70	2.75	1.46	0.00E+00	0.00E+00
PA1818	ldcA	3	Lysine-specific pyridoxal 5'-phosphate (PLP)-dependent carboxylase	1.00	1.00	43.00	18.00	1.00	0.00	2.73	1.45	2.82E-03	3.10E-03
PA3234	ActP	9	Acetate permease (predicted)	1.00	1.00	43.00	23.00	1.00	0.00	2.73	1.45	2.82E-03	3.10E-03
PA4619		3	Predicted iron/heme-binding cytochrome	2.00	3.00	224.00	79.00	3.24	1.70	2.73	1.45	2.31E-07	1.16E-06
PA5040	PilQ	2	Type 4 fimbrial biogenesis outer membrane protein	47.00	71.00	54612.00	62315.00	3.22	1.69	2.58	1.37	0.00E+00	0.00E+00
PA0535	DnaE	5	DnaE (DNA polymerase III alpha subunit)	2.00	2.00	232.00	469.00	4.86	2.28	2.45	1.29	2.27E-04	2.51E-04
PA5041	PilP	2	Type 4 fimbrial biogenesis protein	8.00	13.00	5957.00	6871.00	2.99	1.58	2.43	1.28	0.00E+00	0.00E+00
PA0495	AtzF	3	Allophanate hydrolase subunit 1	1.00	1.00	38.00	38.00	1.00	0.00	2.42	1.27	1.04E-02	1.11E-02

PA3716		16	Protein of unknown function (contains a WG repeat)	1.00	1.00	36.00	13.00	1.00	0.00	2.29	1.19	1.72E-02	1.82E-02
PA5043	PilN	2	Type 4 fimbrial biogenesis protein	6.00	10.00	2363.00	4345.00	2.92	1.54	2.19	1.13	1.30E-12	4.60E-11
PA3620	MutS	10	DNA mismatch repair protein MutS	2.00	1.00	34.00	17.00	9.72	3.28	2.16	1.11	2.81E-02	2.97E-02
PA5044	PilM	2	Type 4 fimbrial biogenesis protein	17.00	31.00	14686.00	15265.00	2.67	1.41	2.11	1.07	0.00E+00	0.00E+00
PA0968	FadM	4	acetyl-CoA thioesterase (Tol receptor operon regulated by iron)	1.00	1.00	33.00	142.00	1.00	0.00	2.10	1.07	3.58E-02	3.78E-02
PA3840		7	rRNA (adenine-N6-)-methyltransferase activity	1.00	1.00	33.00	176.00	1.00	0.00	2.10	1.07	3.58E-02	3.78E-02
PA0027		3	Phosphatidylethanolamine (PE) lipid taxis system (3' to HemF operon)	1.00	2.00	303.00	77.00	2.43	1.28	2.07	1.05	3.73E-03	4.07E-03
PA0106	CoxA	3	Cytochrome C oxidase subunit I	1.00	2.00	325.00	49.00	2.43	1.28	2.07	1.05	3.73E-03	4.07E-03
PA0260	Tle3	8	T6SS putative lipase; α-β hydrolase domain	1.00	2.00	172.00	1025.00	2.43	1.28	2.07	1.05	3.73E-03	4.07E-03
PA0268		13	Aspartate amidotransferease superfamily regulator	1.00	2.00	130.00	73.00	2.43	1.28	2.07	1.05	3.73E-03	4.07E-03
PA0464	CreC	13	Two-component histidine kinase CreC	1.00	2.00	182.00	191.00	2.43	1.28	2.07	1.05	3.73E-03	4.07E-03
PA0509	NirN	3	Heme d1 biosynthesis protein	1.00	2.00	151.00	30.00	2.43	1.28	2.07	1.05	3.73E-03	4.07E-03
PA0625	HylD	9	Type I secretion system protein	1.00	2.00	490.00	276.00	2.43	1.28	2.07	1.05	3.73E-03	4.07E-03
PA0792	PrpD	3	Aconitase activity	1.00	2.00	524.00	137.00	2.43	1.28	2.07	1.05	3.73E-03	4.07E-03
PA0820		3	HNH nuclease or endonuclease	1.00	2.00	933.00	234.00	2.43	1.28	2.07	1.05	3.73E-03	4.07E-03
PA0845	CerN	8	Hydrolyzes ceramide to generate sphingosine and fatty acid; possible exotoxin	1.00	2.00	7349.00	15601.00	2.43	1.28	2.07	1.05	3.73E-03	4.07E-03
PA1069		16	Hypothetical protein	1.00	2.00	274.00	106.00	2.43	1.28	2.07	1.05	3.73E-03	4.07E-03
PA2670		16	Hypothetical protein	1.00	2.00	129.00	43.00	2.43	1.28	2.07	1.05	3.73E-03	4.07E-03
PA2761		16	Predicted glycine/betaine transporter	1.00	2.00	218.00	69.00	2.43	1.28	2.07	1.05	3.73E-03	4.07E-03
PA3053	MhpC	3	Flavoenzyme catalyzing an aromatic hydroxylation and a ring-cleavage reaction	1.00	2.00	249.00	313.00	2.43	1.28	2.07	1.05	3.73E-03	4.07E-03
PA3305		16	Hypothetical protein	1.00	2.00	109.00	144.00	2.43	1.28	2.07	1.05	3.73E-03	4.07E-03
PA3340	FimV-like	2	Twitching motility	1.00	2.00	98.00	168.00	2.43	1.28	2.07	1.05	3.73E-03	4.07E-03
PA3710	BetA	8	Choline dehydrogenase; betaine biosynthesis from choline	1.00	2.00	1982.00	3686.00	2.43	1.28	2.07	1.05	3.73E-03	4.07E-03
PA3898		13	AraC-type transcriptional regulator	1.00	2.00	133.00	335.00	2.43	1.28	2.07	1.05	3.73E-03	4.07E-03
PA4026		3	Gcn5-related N-acetyltransferase (GNAT)	1.00	2.00	4897.00	580.00	2.43	1.28	2.07	1.05	3.73E-03	4.07E-03
PA4113	AraJ	9	Putative arabinose efflux transporter	1.00	2.00	574.00	684.00	2.43	1.28	2.07	1.05	3.73E-03	4.07E-03
PA4625	CrdA	13	c-di-GMP binding, coordinates extracellular matrix, positively regulates biofilms	8.00	16.00	7149.00	2826.00	2.43	1.28	2.07	1.05	0.00E+00	0.00E+00
PA4689	MlaD	8	Lipid transporter component	3.00	6.00	560.00	961.00	2.43	1.28	2.07	1.05	5.07E-07	2.53E-06
PA4733	AcsB	3	Acetyl-CoA synthetase	1.00	2.00	120.00	105.00	2.43	1.28	2.07	1.05	3.73E-03	4.07E-03
PA4913		9	Probable outer membrane amino acid transporter binding protein	1.00	2.00	76.00	1052.00	2.43	1.28	2.07	1.05	3.73E-03	4.07E-03
PA4925	MscS	9	Small-conductance mechanosensitive channel protein	1.00	2.00	712.00	61.00	2.43	1.28	2.07	1.05	3.73E-03	4.07E-03
PA3641	AruH	3	L-Arginine catobolism; pyruvate transaminase	1.00	2.00	3187.00	3288.00	2.43	1.28	2.07	1.05	3.73E-03	4.07E-03
PA5058	PhaC	3	Poly(3-hydroxyalkanoic acid) synthase 2	1.00	2.00	312.00	231.00	2.43	1.28	2.07	1.05	3.73E-03	4.07E-03
PA5217	PotD	15	PotD; Fe3+ and polyamine ABC transporter periplasmic binding protein	1.00	2.00	68.00	121.00	2.43	1.28	2.07	1.05	3.73E-03	4.07E-03
PA5412		16	Hypothetical protein	1.00	2.00	820.00	107.00	2.43	1.28	2.07	1.05	3.73E-03	4.07E-03
PA4587	CcpR	3	Cytochrome c551 peroxidase precursor	1.00	1.00	32.00	583.00	1.00	0.00	2.03	1.02	4.54E-02	4.77E-02
PA4607		16	DUF3015 containing protein of unknown function	1.00	1.00	32.00	248.00	1.00	0.00	2.03	1.02	4.54E-02	4.77E-02
PA4551	PilV	2	Type 4 fimbrial biogenesis protein	7.00	12.00	2631.00	4472.00	2.84	1.50	2.00	1.00	1.37E-11	2.29E-10
PA5523	HemL-like	4	HemL/ArgD, pyridoxal phosphate binding	1.00	2.00	63.00	146.00	2.43	1.28	2.00	1.00	5.78E-03	6.17E-03
PA4675	ChtA	4	Xenosiderophore transporter (TonB-dependent)	2.00	3.00	251.00	210.00	3.24	1.70	1.99	0.99	8.04E-04	8.84E-04
PA2151		3	Zinc-dependent alchohol dehydrogenase	1.00	1.00	31.00	50.00	1.00	0.00	1.97	0.98	5.73E-02	6.01E-02
PA0101		16	HEAT domain repeats	1.00	1.00	30.00	23.00	1.00	0.00	1.91	0.93	7.21E-02	7.56E-02
PA4499	PsdR	13	Regulates dipeptide, aminolevulinic acid and heme transport	1.00	1.00	30.00	723.00	1.00	0.00	1.91	0.93	7.21E-02	7.56E-02
PA5233	FliL	2	Flagellar basal body protein FliL	1.00	1.00	29.00	171.00	1.00	0.00	1.84	0.88	9.03E-02	9.44E-02
PA4099	OprB	9	Carbohydrate-selective porin (glucose activated)	1.00	1.00	28.00	261.00	1.00	0.00	1.78	0.83	1.13E-01	1.18E-01
PA2252		9	Predicted AGCS sodium/alanine/glycine symporter	1.00	2.00	54.00	62.00	2.43	1.28	1.72	0.78	3.67E-02	3.88E-02
PA2563		9	Predicted sulfate transporter	1.00	1.00	27.00	30.00	1.00	0.00	1.72	0.78	1.40E-01	1.46E-01

PA5155		9	Predicted amino acid (lysine/arginine/ornithine/ histidine/octopine) transporter	1.00	1.00	26.00	453.00	1.00	0.00	1.65	0.72	1.72E-01	1.78E-01
PA5181		10	Predicted molybdenum ion / Fe-sulfur cluster binding protein	1.00	1.00	26.00	272.00	1.00	0.00	1.65	0.72	1.72E-01	1.78E-01
PA4526	PilB	2	Type 4 fimbrial biogenesis protein	22.00	52.00	23425.00	46461.00	2.06	1.04	1.62	0.70	0.00E+00	0.00E+00
PA0525	NorD	3	Denitrification protein	1.00	2.00	50.00	47.00	2.43	1.28	1.59	0.67	7.73E-02	8.09E-02
PA1859		13	LysR-type transcriptional regulator	1.00	1.00	25.00	190.00	1.00	0.00	1.59	0.67	2.12E-01	2.19E-01
PA2474		3	Putative MetA-pathway of phenol degradation (in operon with GST)	1.00	1.00	23.00	42.00	1.00	0.00	1.46	0.55	3.13E-01	3.22E-01
PA4227	PchR	4	Binds and regulates pyochelin siderophore synthesis and uptake	1.00	1.00	23.00	42.00	1.00	0.00	1.46	0.55	3.13E-01	3.22E-01
PA4554	PilY1	2	Type 4 fimbrial biogenesis protein	52.00	132.00	82011.00	40384.00	1.91	0.94	1.46	0.54	0.00E+00	0.00E+00
PA1410	PotD	15	Spermidine/putrescine-binding periplasmic protein	1.00	1.00	22.00	59.00	1.00	0.00	1.40	0.48	3.77E-01	3.86E-01
PA3877	Nark1	3	Nitrite metabolism operon	1.00	1.00	22.00	124.00	1.00	0.00	1.40	0.48	3.77E-01	3.86E-01
PA0067	PrlC	3	Oligopeptidase	1.00	3.00	530.00	286.00	1.62	0.70	1.38	0.46	1.45E-01	1.51E-01
PA0158	TriC	9	Detergent/antibiotic efflux pump	1.00	3.00	134.00	130.00	1.62	0.70	1.38	0.46	1.45E-01	1.51E-01
PA0266	GabT	15	Arginine degradation	1.00	3.00	300.00	167.00	1.62	0.70	1.38	0.46	1.45E-01	1.51E-01
PA1027	AmaB	3	Aldehyde dehydrogenase	1.00	3.00	151.00	164.00	1.62	0.70	1.38	0.46	1.45E-01	1.51E-01
PA1383		16	Hypothetical protein	1.00	3.00	561.00	90.00	1.62	0.70	1.38	0.46	1.45E-01	1.51E-01
PA4514	PiuA	4	Outer membrane receptor for iron transport	1.00	3.00	71.00	132.00	1.62	0.70	1.38	0.46	1.45E-01	1.51E-01
PA4985	PotD	15	Fe3+ and/or spermidine/putrescine ABC transporter periplasmic binding protein	1.00	3.00	456.00	197.00	1.62	0.70	1.38	0.46	1.45E-01	1.51E-01
PA5022	MscK	9	Small-conductance mechanosensitive channel protein	1.00	3.00	313.00	563.00	1.62	0.70	1.38	0.46	1.45E-01	1.51E-01
PA5367	PstA	9	Membrane protein component of ABC phosphate transporter	1.00	3.00	89.00	548.00	1.62	0.70	1.38	0.46	1.45E-01	1.51E-01
PA3805	PilF	2	Type 4 fimbrial biogenesis protein	11.00	32.00	6850.00	12411.00	1.67	0.74	1.32	0.40	4.45E-05	1.73E-04
PA4163		7	Predicted Asp-tRNAAsn/Glu-tRNAGln amidotransferase A subunit	1.00	1.00	20.00	69.00	1.00	0.00	1.27	0.35	5.34E-01	5.45E-01
PA4986		3	FAD-dependent oxidoreductase	1.00	1.00	20.00	15.00	1.00	0.00	1.27	0.35	5.34E-01	5.45E-01
PA5327	SphC	3	Sphingosine degradation / FAD - binding oxidoreductase	1.00	1.00	20.00	19.00	1.00	0.00	1.27	0.35	5.34E-01	5.45E-01
PA5372	BetA	3	Choline dehydrogenase; betaine biosynthesis from choline	1.00	3.00	58.00	145.00	1.62	0.70	1.23	0.30	3.58E-01	3.68E-01
PA5516	PdxY	3	Pyridoxamine kinase, salvage pathway of pyridoxal 5'-phosphate (PLP)	1.00	1.00	19.00	148.00	1.00	0.00	1.21	0.27	6.28E-01	6.39E-01
PA4553	PilX	2	Type 4 fimbrial biogenesis protein	6.00	20.00	1555.00	4113.00	1.46	0.54	1.20	0.26	4.15E-02	4.37E-02
PA0426	MexB	9	Resistance-Nodulation-cell Division (RND) multidrug efflux transporter	2.00	4.00	249.00	261.00	2.43	1.28	1.19	0.25	3.69E-01	3.79E-01
PA0142		3	8-oxoguanine deaminase	1.00	2.00	36.00	59.00	2.43	1.28	1.14	0.19	6.29E-01	6.39E-01
PA1838	CysI	3	Sulfite reductase	1.00	1.00	18.00	106.00	1.00	0.00	1.14	0.19	7.33E-01	7.42E-01
PA1858	StrB	3	Streptomycin 3"- kinase	1.00	1.00	18.00	84.00	1.00	0.00	1.14	0.19	7.33E-01	7.42E-01
PA2591	VqsR	1	Quorum sensing regulator (N-acyl homoserine lactones)	1.00	1.00	18.00	163.00	1.00	0.00	1.14	0.19	7.33E-01	7.42E-01
PA2837	OmpA	9	Outer membrane efflux protein	1.00	1.00	18.00	26.00	1.00	0.00	1.14	0.19	7.33E-01	7.42E-01
PA3079		9	Predicted efflux protein	1.00	1.00	18.00	20.00	1.00	0.00	1.14	0.19	7.33E-01	7.42E-01
PA4060		3	S-adenosylmethionine-dependent methyltransferase	1.00	1.00	18.00	220.00	1.00	0.00	1.14	0.19	7.33E-01	7.42E-01
PA4528	PilD	2	Type 4 prepilin peptidase	4.00	12.00	377.00	2589.00	1.62	0.70	1.14	0.19	2.53E-01	2.61E-01
PA2492	MexT	13	Transcriptional regulator	4.00	6.00	341515.00	40398.00	3.24	1.70	1.12	0.17	4.73E-01	4.82E-01
PA4556	PilE	2	Type 4 fimbrial biogenesis protein	5.00	19.00	929.00	5911.00	1.28	0.36	1.09	0.12	3.59E-01	3.69E-01
PA5449	WbpX	3	Glycosyltransferase	1.00	2.00	33.00	102.00	2.43	1.28	1.05	0.07	8.66E-01	8.73E-01
PA0301	SpuE	15	Spermidine/putrescine-binding periplasmic protein	1.00	4.00	99.00	302.00	1.22	0.28	1.03	0.05	8.72E-01	8.77E-01
PA4221	FptA	4	Fe(III)-pyochelin outer membrane receptor	2.00	5.00	105.00	639.00	1.94	0.96	1.03	0.04	8.70E-01	8.76E-01
PA3241	RssA	11	Predicted phospholipase (patatin domain)	1.00	1.00	16.00	143.00	1.00	0.00	1.02	0.02	9.67E-01	9.68E-01
PA3601		7	50S ribosomal protein L31	1.00	1.00	16.00	636.00	1.00	0.00	1.02	0.02	9.67E-01	9.68E-01
PA5047		16	Putative Zn-dependent protease	1.00	1.00	16.00	161.00	1.00	0.00	1.02	0.02	9.67E-01	9.68E-01
PA4527	PilC	2	Pilin protein	10.00	35.00	5190.00	10851.00	1.39	0.47	1.01	0.01	9.07E-01	9.10E-01

* Yellow highlight = genes used for validation in these studies.
Grey highlight = genes with p values below 0.05

** PAO1 locus number

*** Category: 1 = Quorum sensing; 2= Adherence/Motility; 3 = Energy/Metabolism; 4 = Fe/Heme; 5 = DNA; 6 = RNA; 7 = Protein; 8 = Virulence; 9 = Transportation; 10 = Cellular stress; 11= Fatty acids; 12 = Cell wall/Envelope; 13 = Regulation/Signal transduction; 14 = Phage; 15 = Polyamines; 16 = Unknown
Based on manual annotation.

Table S1B. Met-Seq Enriched Promoters

Gene ID*	Name	Category **	Annotation	AveUnique hits (treatment)	Ave. Unique hits (control)	Ave. Raw Reads (treatment)	Ave. Raw Read (control)	Ratio_ Insertions (Treatment / control)	Log2-fold Change (Insertions)	Ratio_ reads (Treatment /control)	Log2-fold Change (reads)	p value (proportions_reads)	Adj. pvalue (proportions_reads)
PA4731	PanD	7	Aspartate 1-decarboxylase precursor	1	0	53	0	15.83	3.98	33.69	5.07	6.46E-09	1.12E-06
PA0051	PhzH	3	Phenazine biosynthesis	1	1	100	248	1.00	0.00	4.13	2.05	5.74E-06	1.10E-04
PA0155	PcaR	13	Aromatic compound degradation	1	1	208	72	1.00	0.00	4.13	2.05	5.74E-06	1.10E-04
PA0473	GST	10	Glutathione-S-transferase	1	1	207	424	1.00	0.00	4.13	2.05	5.74E-06	1.10E-04
PA0946		16	Hypothetical	1	1	110	97	1.00	0.00	4.13	2.05	5.74E-06	1.10E-04
PA0990		3	Nudix hydrolase	1	1	251	42	1.00	0.00	4.13	2.05	5.74E-06	1.10E-04
PA1750	AroG1	3	DHAP synthetase	1	1	68	135	1.00	0.00	4.13	2.05	5.74E-06	1.10E-04
PA1813	GloB	10	Hydroxyacylglutathione hydrolase	1	1	187	17	1.00	0.00	4.13	2.05	5.74E-06	1.10E-04
PA1814	KerV	3	Methyltransferase	1	1	187	17	1.00	0.00	4.13	2.05	5.74E-06	1.10E-04
PA1943	RimI	13	Acetyltransferase	1	1	89	30	1.00	0.00	4.13	2.05	5.74E-06	1.10E-04
PA2548		12	Periplasmic binding OmpA-like	1	1	276	342	1.00	0.00	4.13	2.05	5.74E-06	1.10E-04
PA2897	MocR-like	13	Transcription factor/PLP-dependent aminotransferase	1	1	911	92	1.00	0.00	4.13	2.05	5.74E-06	1.10E-04
PA3340	FimV	2	Motility	1	1	543	46	1.00	0.00	4.13	2.05	5.74E-06	1.10E-04
PA4234	Ssb	5	Replication, single stranded DNA binding	1	1	65	218	1.00	0.00	4.13	2.05	5.74E-06	1.10E-04
PA4574	YqhA	16	Uncharacterized membrane protein	1	1	584	99	1.00	0.00	4.13	2.05	5.74E-06	1.10E-04
PA4876	OsmE	12	Osmotically-inducible lipoprotein	1	1	2113	281	1.00	0.00	4.13	2.05	5.74E-06	1.10E-04
PA5461		16	Hypothetical; DUF2388	1	1	191	49	1.00	0.00	4.13	2.05	5.74E-06	1.10E-04
PA5566		16	Hypothetical	1	1	643	42	1.00	0.00	4.13	2.05	5.74E-06	1.10E-04
PA3921	MalT	13	Transcription factor	1	1	43	36	1.00	0.00	2.73	1.45	2.82E-03	2.89E-03
PA3577		16	Hypothetical	1	1	40	22	1.00	0.00	2.54	1.35	6.21E-03	6.21E-03
PA2754	ElaB	7	Membrane-anchored ribosome-binding protein	1	2	255	154	3.96	1.98	2.07	1.05	3.73E-03	3.79E-03
PA2755	Eco	8	Ecotin precursor	1	2	121	65	3.96	1.98	2.07	1.05	3.73E-03	3.79E-03
PA4524.1		7	tRNA	1	2	1072	3123	3.96	1.98	2.07	1.05	3.73E-03	3.79E-03

* PAO1 locus number

** Category: 1 = Quorum sensing; 2= Adherence/Motility; 3 = Energy/Metabolism; 4 = Fe/Heme; 5 = DNA; 6 = RNA; 7 = Protein; 8 = Virulence; 9 = Transportation; 10 = Cellular 11= Fatty acids; 12 = Cell wall/Envelope; 13 = Regulation/Signal transduction; 14 = Phage; 15 = Polyamines; 16 = Unknown

Table S1C.Met-Seq Enriched sRNAs

Gene ID*	sRNA ID	sRNA start_end	sRNA length	Intergenic start_end	Inter-genic length	Name	Category **	Annotation	AveUnique hits (treatment)	Ave. Unique hits (control)	Ave. Raw Reads (treatment)	Ave. Raw Read (control)	Ratio_ Insertions (Treatment /control)	Log2-fold Change (Insertions)	Ratio_ reads (Treatment /control)	Log2-fold Change (reads)	pvalue (proportions_ reads)	Adj. pvalue (proportions_reads)
PA5042	as223	5678557_ 5678652	96	na	na	*pilO*	2	Type 4 fimbrial biogenesis protein PilO	2	2	1527	1818	5.48	2.45	4.13	2.05	1.41E-10	5.29E-09
PA5058	as226	5699012_ 5699062	51	na	na	*phaC 2*	3	Poly(3-hydroxyalkanoic acid) synthase 2	1	1	312	180	1.00	0.00	4.13	2.05	5.74E-06	8.61E-05
PA2906_ PA2907	pant272	3261207_ 3261426	114	3261186_ 3261521	335	*cobG*	?	Colbamin biosynthesis (RNA regulation)	1	1	65	104	1.00	0.00	4.13	2.05	5.74E-06	8.61E-05
PA3433_ PA3434	pant317	3841795_ 3842086	292	3841738_ 3842273	535		?	LysR-type TF/Transposase	1	1	226	175	1.00	0.00	4.13	2.05	5.74E-06	8.61E-05
PA3993_ PA3994	pant368	4474657_ 4474804	148	4474640_ 4474958	318		?	Predicted alpha/beta hydrolase/Transposase	1	1	85	61	1.00	0.00	4.13	2.05	5.74E-06	8.61E-05
PA3577_ PA3578	pant335	4009052_ 4009156	105	4009052_ 4009541	491	*phzC/ F*	?	Shikimate/chorismate biosythesis pathway	1	1	40	22	1.00	0.00	2.54	1.35	6.21E-03	6.30E-03
PA0495	as22	554963_5 55078	116	na	na	*atzF*	3	Allophanate hydrolase subunit 1	1	1	38	38	1.00	0.00	2.42	1.27	1.04E-02	1.04E-02
PA4554	as197	5101107_ 5101200	94	na	na	*pilY1*	2	Pilus	5	10	58588	5160	2.74	1.45	2.07	1.05	8.82E-11	6.61E-09
PA2319_ PA2320	pant213	2557983_ 2558363	381	2557965_ 2558917	952	*gntR*	?	regluator of glucose/gluconate metabolism	1	2	65	92	2.74	1.45	2.07	1.05	3.73E-03	3.88E-03
PA5190_ PA5191	pant479	5843826_ 5843965	140	5843801 _5844053	252	NfnB/ LpfA	?	NADPH quinone oxidoreductase/polar fimbrial protein	1	2	110	578	2.74	1.45	2.07	1.05	3.73E-03	3.88E-03
PA4554	as198	5101511_ 5101611	101	na	na	*pilY1*	2	Pilus	3	8	854	3007	2.05	1.04	1.49	0.58	2.47E-03	2.61E-03

* PAO1 locus number
** Category: 1 = Quorum sensing; 2= Adherence/Motility; 3 = Energy/Metabolism; 4 = Fe/Heme; 5 = DNA; 6 = RNA; 7 = Protein; 8 = Virulence; 9 = Transportati
11= Fatty acids; 12 = Cell wall/Envelope; 13 = Regulation/Signal transduction; 14 = Phage; 15 = Polyamines; 16 = Unknown
