# Supplementary material for: A High-Throughput Method for Identifying Novel Genes That Influence Metabolic Pathways Reveals New Iron and Heme Regulation in Pseudomonas aeruginosa
Source: mSystems. 2021 Feb 2;6(1):e00933-20. doi: 10.1128/mSystems.00933-20 (PMC7857532; doi:10.1128/mSystems.00933-20)
Supplement: TABLE S2 [file mSystems.00933-20-st002.pdf]

Table S2A. GO Enrichments						
**ID**	**Gene set Name**	**# Gene set size**	**# MetSeq genes**	**pvalue (hypergeometric)**	**Fraction of gene set**	**Genes***
GO:0022857	Transmembrane transporter activity	185	13	0.003672607	0.07027027	PA0158,PA0426,PA1108,PA1247,PA2837,PA2925,PA3234,PA3305,PA4187,PA4804,PA5155,PA5378,PA5434
GO:0020037	Heme binding	60	7	0.000822336	0.116666667	PA0106,PA0509,PA1838,PA1941,PA4587,PA4613,PA4619
GO:0038023	signaling receptor activity	12	3	0.000502291	0.25	PA4221,PA4514,PA4675
GO:0016614	Oxidoreductase activity	13	3	0.000706505	0.230769231	PA3710,PA4619,PA5372
GO:0004222	Metalloendopeptidase activity	16	3	0.001660859	0.1875	PA0067,PA0277,PA5047
GO:0051537	2 iron, 2 sulfur cluster binding	23	3	0.006718649	0.130434783	PA0792,PA4904,PA4905
GO:0006352	DNA-templated transcription, initiation	25	3	0.009107436	0.12	PA0149,PA1455,PA2896

*PAO1 locus number

Table S2B. KEGG Enrichments

ID	Gene set Name	# Gene set size	# MetSeq genes	pvalue (hypergeometric)	Fraction of gene set	Genes*
pae02024	Quorum sensing	95	6	0.040612871	0.0631579	PA0652,PA1001,PA2843,PA4804, PA4913,PA4985
pae00330	Arginine and proline metabolism	44	4	0.015492103	0.0909091	PA1027,PA1818,PA4163,PA4908, PA4976
pae00640	Propanoate metabolism	47	4	0.020184082	0.0851064	PA0747,PA0792,PA0887,PA4733
pae00627	Aminobenzoate degradation	17	3	0.00211518	0.1764706	PA4163,PA4904,PA4905
pae01503	Cationic antimicrobial peptide (CAMP) resistance	28	3	0.013628512	0.1071429	PA0426,PA0920,PA1179
pae00010	Glycolysis / Gluconeogenesis	37	3	0.03479919	0.0810811	PA0887,PA1027,PA4733
pae00380	Tryptophan metabolism	27	3	0.011990809	0.1111111	PA1027,PA4163,PA4613

*PAO1 locus number

Table S2C. Cytoscape Enrichments

Largest cluster (lower right)

Cluster 1 **Transcriptional regulation**

KEGG

ID	Gene set Name	Gene set size	DE genes	Pvalue (hypergeometric)	Genes*
pae02020	Two-component system	200	7	2.14535E-06	PA0463,PA0464,PA0652,PA0920, PA1179,PA1455,PA3349

GO

ID	Gene set Name	Gene set size	DE genes	Pvalue (hypergeometric)	Genes
GO:0003677	DNA binding	337	5	0.003835118	PA0463,PA0652,PA1179,PA1455,
GO:0006355	regulation of transcription, DNA-templated	358	4	0.022850285	PA0463,PA0652,PA1179,PA2896
GO:0003700	DNA-binding transcription factor activity	278	3	0.038101909	PA0652,PA1455,PA2896
GO:0000160	phosphorelay signal transduction system	99	3	0.001038175	PA0463,PA1179,PA3349

Cluster 2 **Transmembrane transport**

GO

ID	Gene set Name	Gene set size	DE genes	Pvalue (hyper)	Genes
GO:0003677	DNA binding	337	3	0.040463391	PA0149,PA3898,PA4227
GO:0022857	transmembrane transporter activity	185	3	0.00538199	PA0158,PA0426,PA2837
GO:0055085	transmembrane transport	356	4	1.10E-02	PA0158,PA0426,PA2837,PA5219
GO:0003700	DNA-binding transcription factor activity	278	4	0.003895206	PA0149,PA2492,PA3898,PA4227

Cluster 3 **Metabolism**

KEGG

ID	Gene set Name	Gene set size	DE genes	Pvalue (hyper)	Genes
pae01130	Biosynthesis of antibiotics	247	5	2.5509E-05	PA0887,PA1001,PA1027,PA3517, PA4733
pae01110	Biosynthesis of secondary metabolites	338	5	0.000149396	PA0887,PA1001,PA1027,PA3517, PA4733
pae01200	Carbon metabolism	129	3	0.000307061	PA0747,PA0887,PA4733
pae00010	Glycolysis / Gluconeogenesis	37	3	2.13259E-06	PA0887,PA1027,PA4733
pae01100	Metabolic pathways	800	8	5.50556E-05	PA0266,PA0747,PA0887,PA1001, PA1027,PA3517,PA4733,PA5093
pae01120	Microbial metabolism in diverse environments	265	6	2.35786E-06	PA0266,PA0887,PA1027,PA4733, PA4904,PA4905
pae00640	Propanoate metabolism	47	4	9.68E-08	PA0747,PA0792,PA0887,PA4733
pae00620	Pyruvate metabolism	61	3	1.62207E-05	PA0887,PA1027,PA4733

GO

ID	Gene set Name	Gene set size	DE genes	Pvalue (hyper)	Genes
GO:0003824	catalytic activity	421	6	5.04981E-05	PA0266,PA0268,PA0887,PA3517, PA4733,PA5093
GO:0009058	biosynthetic process	78	3	4.31548E-05	PA0268,PA1001,PA1758
GO:0055114	oxidation-reduction process	445	4	0.004864774	PA0747,PA1027,PA4904,PA4905
GO:0016491	oxidoreductase activity	281	4	0.000622253	PA0747,PA1027,PA4904,PA4905
GO:0051537	2 iron, 2 sulfur cluster binding	23	3	2.92E-07	PA0792,PA4904,PA4905

Cluster 4 **Heme binding**

GO

ID	Gene set Name	Gene set size	DE genes	Pvalue (hyper)	Genes
GO:0009055	electron transfer activity	82	3	6.93238E-05	PA1941,PA4587,PA4619
GO:0020037	heme binding	60	5	8.90E-09	PA0106,PA1838,PA1941,PA4587, PA4619
GO:0055114	oxidation-reduction process	445	4	0.006619296	PA0106,PA1838,PA4587,PA5181
GO:0016491	oxidoreductase activity	281	3	0.007116981	PA1838,PA4587,PA5181

*PAO1 locus number

Table S2D. DAVID Enrichments

**Annotation Cluster 1**	**Enrichment Score: 1.489300855428664**												
**Category**	**Term**	**Count**	**%**	**PValue**	**Genes***	**List Total**	**Pop Hits**	**Pop Total**	**Fold Enrich-ment**	**Bonferroni**	**Benjamini**	**FDR**
UP_SEQ_ FEATURE	signal peptide	8	4.30	0.01	PA4221, PA1169, PA2755, PA0845, PA4587, PA5040, PA5217, PA4613	31	101	1220	3.12	0.48	0.28	8.95
GOTERM_ CC_DIRECT	GO:0042597~periplasmic space	7	3.76	0.03	PA1410, PA1169, PA2755, PA4587, PA5378, PA4613, PA0301	94	69	2644	2.85	0.53	0.53	23.03
UP_ KEYWORDS	Periplasm	5	2.69	0.12	PA1169, PA2755, PA4587, PA4613, PA0301	185	56	5494	2.65	1.00	0.84	74.05

**Annotation Cluster 2**	**Enrichment Score: 1.1050330157086925**											
**Category**	**Term**	**Count**	**%**	**PValue**	**Genes**	**List Total**	**Pop Hits**	**Pop Total**	**Fold Enrichment**	**Bonferroni**	**Benjamini**	**FDR**
GOTERM_ MF_DIRECT	GO:0020037~heme binding	7	3.76	0.02	PA4619, PA1838, PA4587, PA1941, PA0509, PA0106, PA4613	125	64	3597	3.15	0.94	0.94	22.55
UP_ KEYWORDS	Iron	9	4.84	0.12	PA4619, PA4221, PA1169, PA1838, PA4587, PA4904, PA4905, PA0106, PA4613	185	148	5494	1.81	1.00	0.77	75.76
UP_ KEYWORDS	Heme	4	2.15	0.18	PA4619, PA4587, PA0106, PA4613	185	44	5494	2.70	1.00	0.80	88.66

**Annotation Cluster 3**	**Enrichment Score: 1.0150459145791382**											
**Category**	**Term**	**Count**	**%**	**PValue**	**Genes**	**List Total**	**Pop Hits**	**Pop Total**	**Fold Enrichment**	**Bonferroni**	**Benjamini**	**FDR**
INTERPRO	IPR014710: RmlC-like jelly roll fold	6	3.23	0.04	PA0535, PA0652, PA2762, PA4499, PA0527, PA1140	169	55	4955	3.20	1.00	1.00	40.75
INTERPRO	IPR013096: Cupin 2, conserved barrel	3	1.61	0.09	PA0535, PA4499, PA1140	169	15	4955	5.86	1.00	1.00	72.46
INTERPRO	IPR011051: RmlC-like cupin domain	4	2.15	0.27	PA0535, PA2762, PA4499, PA1140	169	53	4955	2.21	1.00	1.00	98.58

**Annotation Cluster 4**	**Enrichment Score: 1.004471094419736**											
**Category**	**Term**	**Count**	**%**	**PValue**	**Genes**	**List Total**	**Pop Hits**	**Pop Total**	**Fold Enrichment**	**Bonferroni**	**Benjamini**	**FDR**
GOTERM_ MF_DIRECT	GO:0020037~heme binding	7	3.76	0.02	PA4619, PA1838, PA4587, PA1941, PA0509, PA0106, PA4613	125	64	3597	3.15	0.94	0.94	22.55
INTERPRO	IPR009056: Cytochrome c domain	4	2.15	0.10	PA4619, PA4587, PA1941, PA0509	169	33	4955	3.55	1.00	1.00	76.23
GOTERM_ MF_DIRECT	GO:0009055~electron carrier activity	6	3.23	0.45	PA4619, PA5401, PA4587, PA1941, PA4905, PA0509	125	127	3597	1.36	1.00	1.00	99.90

**Annotation Cluster 5**	**Enrichment Score: 0.9557228528500402**											
**Category**	**Term**	**Count**	**%**	**PValue**	**Genes**	**List Total**	**Pop Hits**	**Pop Total**	**Fold Enrichment**	**Bonferroni**	**Benjamini**	**FDR**

UP_ KEYWORDS	Transmembrane	48	25.81	0.08	PA4113, PA0319, PA0765, PA1108, PA4689, PA5155, PA5367, PA5113, PA4925, PA3877, PA0106, PA1708, PA2563, PA1963, PA3641, PA4804, PA1941, PA3061, PA3340, PA0281, PA2252, PA5412, PA0278, PA4187, PA4553, PA5517, PA4556, PA0426, PA5022, PA0158, PA3079, PA2837, PA5042, PA3234, PA5043, PA5434, PA0920, PA4221, PA4527, PA1247, PA5477, PA4099, PA4551, PA2925, PA0464, PA5219, PA3305, PA4528	185	1161	5494	1.23	1.00	0.85	60.96
UP_ KEYWORDS	Membrane	52	27.96	0.09	PA4113, PA1108, PA3877, PA4514, PA1963, PA4804, PA3340, PA5412, PA5517, PA4553, PA4554, PA4556, PA0158, PA5022, PA2837, PA0920, PA4221, PA4527, PA1247, PA4551, PA2925, PA5219, PA3305, PA4528, PA0319, PA0765, PA4689, PA5155, PA5367, PA5113, PA4925, PA0106, PA1708, PA2563, PA3641, PA1941, PA3061, PA0281, PA2252, PA4675, PA0278, PA4187, PA0426, PA5040, PA3079, PA5042, PA3234, PA5043, PA5434, PA5477, PA4099, PA0464	185	1281	5494	1.21	1.00	0.82	64.35
UP_ KEYWORDS	Transmembrane helix	45	24.19	0.14	PA4113, PA0319, PA0765, PA1108, PA4689, PA5155, PA5367, PA5113, PA4925, PA3877, PA0106, PA1708, PA2563, PA1963, PA3641, PA4804, PA1941, PA3061, PA3340, PA0281, PA2252, PA5412, PA0278, PA4187, PA5517, PA4553, PA4556, PA0426, PA5022, PA0158, PA3079, PA5042, PA3234, PA5043, PA5434, PA0920, PA4527, PA1247, PA5477, PA4551, PA2925, PA0464, PA5219, PA3305, PA4528	185	1125	5494	1.19	1.00	0.78	80.58

GOTERM_ CC_DIRECT	GO:0016021~integral component of membrane	44	23.66	0.14	PA4113, PA0319, PA0765, PA1108, PA4689, PA5155, PA5113, PA4925, PA3877, PA0106, PA1708, PA4514, PA1963, PA3641, PA1941, PA3061, PA3340, PA4137, PA0281, PA2252, PA5412, PA0278, PA4187, PA5517, PA4553, PA4556, PA0426, PA5022, PA0158, PA3079, PA2837, PA5042, PA5043, PA0920, PA4221, PA4527, PA1247, PA5477, PA4551, PA2925, PA0464, PA3305, PA5219, PA4528	94	1067	2644	1.16	0.97	0.83	70.47

Annotation Cluster 6 **Enrichment Score: 0.7302266828369305**

Category	Term	Count	%	PValue	Genes	List Total	Pop Hits	Pop Total	Fold Enrichment	Bonferroni	Benjamini	FDR
INTERPRO	IPR007627: RNA polymerase sigma-70 region 2	3	1.61	0.17	PA0149, PA2896, PA1455	169	22	4955	4.00	1.00	1.00	92.21
UP_ KEYWORDS	Sigma factor	3	1.61	0.18	PA0149, PA2896, PA1455	185	23	5494	3.87	1.00	0.83	88.23
INTERPRO	IPR013325:RNA polymerase sigma factor, region 2	3	1.61	0.18	PA0149, PA2896, PA1455	169	23	4955	3.82	1.00	1.00	93.64
INTERPRO	IPR013324:RNA polymerase sigma factor, region 3/4	3	1.61	0.18	PA0149, PA2896, PA1455	169	23	4955	3.82	1.00	1.00	93.64
INTERPRO	IPR014284:RNA polymerase sigma-70 like domain	3	1.61	0.18	PA0149, PA2896, PA1455	169	23	4955	3.82	1.00	1.00	93.64
GOTERM_ MF_DIRECT	GO:0016987~sigma factor activity	3	1.61	0.23	PA0149, PA2896, PA1455	125	26	3597	3.32	1.00	0.99	94.84

Annotation Cluster 7 **Enrichment Score: 0.6370721288423282**

Category	Term	Count	%	PValue	Genes	List Total	Pop Hits	Pop Total	Fold Enrichment	Bonferroni	Benjamini	FDR
INTERPRO	IPR010105:TonB-dependent siderophore receptor	3	1.61	0.12	PA4675, PA4221, PA4514	169	18	4955	4.89	1.00	1.00	83.35
GOTERM_ MF_DIRECT	GO:0005506~iron ion binding	6	3.23	0.13	PA4619, PA4675, PA4221, PA4514, PA4904, PA0106	125	79	3597	2.19	1.00	0.98	81.44
GOTERM_ MF_DIRECT	GO:0015344~siderophore uptake transmembrane transporter activity	3	1.61	0.20	PA4675, PA4221, PA4514	125	24	3597	3.60	1.00	0.99	92.47
GOTERM_ CC_DIRECT	GO:0009279~cell outer membrane	6	3.23	0.22	PA4675, PA4221, PA4514, PA4099, PA5040, PA2837	94	92	2644	1.83	1.00	0.85	86.15
UP_ KEYWORDS	Receptor	3	1.61	0.23	PA4675, PA4221, PA4514	185	27	5494	3.30	1.00	0.83	94.00
UP_ KEYWORDS	TonB box	3	1.61	0.29	PA4675, PA4221, PA4514	185	32	5494	2.78	1.00	0.89	97.61
INTERPRO	IPR012910:TonB-dependent receptor, plug	3	1.61	0.33	PA4675, PA4221, PA4514	169	35	4955	2.51	1.00	1.00	99.62

INTERPRO	IPR000531:TonB-dependent receptor, beta-barrel	3	1.61	0.33	PA4675, PA4221, PA4514	169	35	4955	2.51	1.00	1.00	99.62
GOTERM_MF_DIRECT	GO:0004872~receptor activity	3	1.61	0.34	PA4675, PA4221, PA4514	125	35	3597	2.47	1.00	1.00	99.22

Annotation Cluster 8 **Enrichment Score: 0.5859278366778857**

Category	Term	Count	%	PValue	Genes	List Total	Pop Hits	Pop Total	Fold Enrichment	Bonferroni	Benjamini	FDR
INTERPRO	IPR015421:Pyridoxal phosphate-dependent transferase, major region, subdomain 1	5	2.69	0.13	PA0268, PA0266, PA1818, PA5523, PA3798	169	58	4955	2.53	1.00	1.00	85.72
INTERPRO	IPR015424:Pyridoxal phosphate-dependent transferase	5	2.69	0.14	PA0268, PA0266, PA1818, PA5523, PA3798	169	59	4955	2.48	1.00	1.00	87.04
INTERPRO	IPR015422:Pyridoxal phosphate-dependent transferase, major region, subdomain 2	4	2.15	0.23	PA0266, PA1818, PA5523, PA3798	169	49	4955	2.39	1.00	1.00	97.23
UP_KEYWORDS	Aminotransferase	3	1.61	0.30	PA0266, PA5523, PA3798	185	33	5494	2.70	1.00	0.88	98.03
GOTERM_MF_DIRECT	GO:0030170~pyridoxal phosphate binding	4	2.15	0.43	PA0268, PA0266, PA5523, PA3798	125	69	3597	1.67	1.00	1.00	99.85
UP_KEYWORDS	Pyridoxal phosphate	3	1.61	0.55	PA0266, PA1818, PA5523	185	55	5494	1.62	1.00	0.92	99.98

Annotation Cluster 9 **Enrichment Score: 0.3580117097059154**

Category	Term	Count	%	PValue	Genes	List Total	Pop Hits	Pop Total	Fold Enrichment	Bonferroni	Benjamini	FDR
INTERPRO	IPR020846:Major facilitator superfamily domain	5	2.69	0.36	PA4113, PA1108, PA4187, PA5219, PA3877	169	89	4955	1.65	1.00	1.00	99.76
INTERPRO	IPR011701:Major facilitator superfamily	4	2.15	0.45	PA4113, PA1108, PA5219, PA3877	169	73	4955	1.61	1.00	1.00	99.97
GOTERM_BP_DIRECT	GO:0055085~transmembrane transport	5	2.69	0.52	PA1247, PA1108, PA5219, PA5022, PA3877	94	119	2950	1.32	1.00	1.00	99.97

Annotation Cluster 10 **Enrichment Score: 0.3271039811625676**

Category	Term	Count	%	PValue	Genes	List Total	Pop Hits	Pop Total	Fold Enrichment	Bonferroni	Benjamini	FDR
UP_SEQ_FEATURE	topological domain:Cytoplasmic	3	1.61	0.22	PA1247, PA0426, PA4528	31	36	1220	3.28	1.00	1.00	92.55
UP_KEYWORDS	Cell inner membrane	8	4.30	0.62	PA4113, PA2252, PA4527, PA1247, PA3641, PA0426, PA4528, PA5434	185	221	5494	1.08	1.00	0.94	100.00
UP_SEQ_FEATURE	transmembrane region	4	2.15	0.77	PA4113, PA1247, PA0426, PA4528	31	158	1220	1.00	1.00	1.00	100.00

Annotation Cluster 11 **Enrichment Score: 0.3267667414274468**

Category	Term	Count	%	PValue	Genes	List Total	Pop Hits	Pop Total	Fold Enrichment	Bonferroni	Benjamini	FDR
KEGG_PATHWAY	pae00010:Glycolysis / Gluconeogenesis	3	1.61	0.31	PA4733, PA0887, PA1027	53	37	1705	2.61	1.00	0.95	97.53

KEGG_ PATHWAY	pae00620:Pyruvate metabolism	3	1.61	0.55	PA4733, PA0887, PA1027	53	60	1705	1.61	1.00	0.99	99.97
KEGG_ PATHWAY	pae01130:Biosynthesis of antibiotics	8	4.30	0.60	PA2843, PA3751, PA4733, PA0887, PA3517, PA1027, PA4613, PA4908	53	238	1705	1.08	1.00	0.99	99.99

Annotation Cluster 12 **Enrichment Score: 0.32593365203775954**

Category	Term	Count	%	PValue	Genes	List Total	Pop Hits	Pop Total	Fold Enrichment	Bonferroni	Benjamini	FDR
UP_ KEYWORDS	Transport	16	8.60	0.21	PA4113, PA2252, PA4587, PA5155, PA0426, PA5040, PA0106, PA5434, PA0301, PA4221, PA4527, PA4526, PA3641, PA1247, PA4099, PA2925	185	354	5494	1.34	1.00	0.82	91.74
UP_ KEYWORDS	Cell membrane	13	6.99	0.53	PA4113, PA2252, PA5155, PA0426, PA5367, PA3234, PA0106, PA5434, PA4527, PA3641, PA1247, PA2925, PA4528	185	356	5494	1.08	1.00	0.92	99.98
UP_ KEYWORDS	Cell inner membrane	8	4.30	0.62	PA4113, PA2252, PA4527, PA1247, PA3641, PA0426, PA4528, PA5434	185	221	5494	1.08	1.00	0.94	100.00
GOTERM_ CC_DIRECT	GO:0005886~plasma membrane	14	7.53	0.74	PA4113, PA2252, PA5155, PA4187, PA5378, PA0426, PA0106, PA4527, PA3641, PA1247, PA2925, PA3305, PA4528, PA0281	94	419	2644	0.94	1.00	0.99	100.00

Annotation Cluster 13 **Enrichment Score: 0.32324867294498294**

Category	Term	Count	%	PValue	Genes	List Total	Pop Hits	Pop Total	Fold Enrichment	Bonferroni	Benjamini	FDR
INTERPRO	IPR011991:Winged helix-turn-helix DNA-binding domain	15	8.06	0.16	PA4769, PA0268, PA0149, PA1141, PA0491, PA1455, PA2591, PA2492, PA1859, PA0652, PA1179, PA0463, PA0527, PA2896, PA5382	169	308	4955	1.43	1.00	1.00	90.71
GOTERM_ MF_DIRECT	GO:0003700~transcription factor activity, sequence-specific DNA binding	13	6.99	0.41	PA4769, PA0268, PA2492, PA3898, PA1859, PA0652, PA0149, PA4227, PA1141, PA2896, PA5382, PA1455, PA0491	125	316	3597	1.18	1.00	1.00	99.77
UP_ KEYWORDS	DNA-binding	18	9.68	0.41	PA4769, PA0268, PA3898, PA0149, PA4227, PA5348, PA1141, PA2591, PA0491, PA1455, PA1859, PA0652, PA1179, PA3620, PA0463, PA0527, PA2896, PA5382	185	471	5494	1.13	1.00	0.92	99.67

GOTERM_ MF_DIRECT	GO:0003677~DNA binding	14	7.53	0.45	PA4769, PA0268, PA0149, PA5348, PA1141, PA0491, PA1455, PA2591, PA0652, PA1179, PA0463, PA0527, PA2896, PA5382	125	354	3597	1.14	1.00	0.99	99.90
UP_ KEYWORDS	Transcription regulation	16	8.60	0.47	PA4769, PA0268, PA3898, PA0149, PA4227, PA1141, PA2591, PA0491, PA1455, PA1859, PA0652, PA1179, PA0463, PA0527, PA2896, PA5382	185	429	5494	1.11	1.00	0.92	99.90
UP_ KEYWORDS	Transcription	16	8.60	0.49	PA4769, PA0268, PA3898, PA0149, PA4227, PA1141, PA2591, PA0491, PA1455, PA1859, PA0652, PA1179, PA0463, PA0527, PA2896, PA5382	185	435	5494	1.09	1.00	0.92	99.94
GOTERM_ BP_DIRECT	GO:0006355~regulation of transcription, DNA-templated	9	4.84	0.57	PA2492, PA3898, PA1179, PA4227, PA0463, PA0527, PA1141, PA1455, PA2591	94	256	2950	1.10	1.00	1.00	99.99
INTERPRO	IPR005119:LysR, substrate-binding	5	2.69	0.59	PA2492, PA1859, PA1141, PA5382, PA0491	169	121	4955	1.21	1.00	1.00	100.00
INTERPRO	IPR000847:Transcription regulator HTH, LysR	5	2.69	0.60	PA2492, PA1859, PA1141, PA5382, PA0491	169	122	4955	1.20	1.00	1.00	100.00
GOTERM_ BP_DIRECT	GO:0006351~transcription, DNA-templated	13	6.99	0.64	PA4769, PA0268, PA3898, PA1859, PA0652, PA1179, PA4227, PA0463, PA1141, PA0527, PA2591, PA5382, PA0491	94	406	2950	1.00	1.00	1.00	100.00
COG_ ONTOLOGY	Transcription	7	3.76	0.78	PA4769, PA2492, PA3898, PA1859, PA1141, PA0491, PA2591	57	174	1308	0.92	1.00	0.99	100.00

Annotation Cluster 14 **Enrichment Score: 0.20812930935648397**

Category	Term	Count	%	PValue	Genes	List Total	Pop Hits	Pop Total	Fold Enrichment	Bonferroni	Benjamini	FDR
UP_SEQ_ FEATURE	nucleotide phosphate-binding region:ATP	5	2.69	0.23	PA1192, PA4526, PA3751, PA5516, PA3620	31	101	1220	1.95	1.00	0.99	93.61
UP_ KEYWORDS	Magnesium	6	3.23	0.47	PA1001, PA3751, PA0845, PA4733, PA0887, PA5516	185	134	5494	1.33	1.00	0.93	99.89
UP_ KEYWORDS	Nucleotide-binding	11	5.91	0.91	PA1192, PA4526, PA3751, PA0652, PA4733, PA0887, PA3061, PA5516, PA4724, PA3620, PA0464	185	425	5494	0.77	1.00	1.00	100.00
UP_ KEYWORDS	ATP-binding	9	4.84	0.93	PA1192, PA4526, PA3751, PA4733, PA0887, PA5516, PA4724, PA3620, PA0464	185	365	5494	0.73	1.00	1.00	100.00

GOTERM_ MF_DIRECT	GO:0005524~ATP binding	9	4.84	0.99	PA1192, PA4526, PA3751, PA4733, PA0887, PA5516, PA4724, PA3620, PA0464	125	426	3597	0.61	1.00	1.00	100.00

**Annotation Cluster 15**	**Enrichment Score: 0.14994127866339493**											
**Category**	**Term**	**Count**	**%**	**PValue**	**Genes**	**List Total**	**Pop Hits**	**Pop Total**	**Fold Enrichment**	**Bonferroni**	**Benjamini**	**FDR**
KEGG_ PATHWAY	pae02020:Two-component system	7	3.76	0.49	PA0920, PA3349, PA0652, PA1179, PA0464, PA0463, PA1455	53	183	1705	1.23	1.00	0.99	99.88
UP_ KEYWORDS	Two-component regulatory system	3	1.61	0.68	PA1179, PA0464, PA0463	185	69	5494	1.29	1.00	0.96	100.00
GOTERM_ BP_DIRECT	GO:0000160~phosphorelay signal transduction system	3	1.61	0.70	PA3349, PA1179, PA0463	94	76	2950	1.24	1.00	1.00	100.00
SMART	SM00448:REC	3	1.61	0.70	PA3349, PA1179, PA0463	30	88	1071	1.22	1.00	1.00	100.00
GOTERM_ CC_DIRECT	GO:0005622~intracellular	6	3.23	0.80	PA3349, PA0652, PA4986, PA1179, PA0464, PA0463	94	187	2644	0.90	1.00	0.99	100.00
INTERPRO	IPR001789:Signal transduction response regulator, receiver domain	3	1.61	0.82	PA3349, PA1179, PA0463	169	90	4955	0.98	1.00	1.00	100.00
INTERPRO	IPR011006:CheY-like superfamily	3	1.61	0.83	PA3349, PA1179, PA0463	169	93	4955	0.95	1.00	1.00	100.00

*PAO1 locus number
