# Supplementary material for: A High-Throughput Method for Identifying Novel Genes That Influence Metabolic Pathways Reveals New Iron and Heme Regulation in Pseudomonas aeruginosa
Source: mSystems. 2021 Feb 2;6(1):e00933-20. doi: 10.1128/mSystems.00933-20 (PMC7857532; doi:10.1128/mSystems.00933-20)
Supplement: TABLE S3 [file mSystems.00933-20-st003.pdf]

Table S3. Strains, plasmids and oligos used in this study.

Strain/Plasmid	Description	Source
***E. coli* strains**		
DH5α	Cloning and plasmid maintenance strain	Lab stock
XL1-Blue	Cloning and plasmid maintenance strain	Lab stock
CC118 λpir	Expresses the λpir protein, allowing replication of suicide plasmids with the R6K origin of replication.	A. Filloux lab stock
SM10 λpir	*P. aeruginosa* conjugation donor strain<br>Expresses the λpir protein, allowing replication of suicide plasmids with the R6K origin of replication.<br>Km^R	A. Filloux lab stock (Simon *et al.*, 1983)
T7 Express *lysY*$^{i/q}$	Protein expression strain.	New England Biolabs, Ipswich, MA
T7 Express *lysY*$^{i/q}$ PA0709	Protein expression strain harbouring pET15DG1 PA0709	This study
T7 Express *lysY*$^{i/q}$ PA3390	Protein expression strain harbouring pET15DG1 PA3390	This study
***P. aeruginosa* strains**		
WT MPAO1	Wild-type *P. aeruginosa* PAO1 strain (clinical, non-respiratory isolate)	Manoil Lab. (Holloway, 1955; Jacobs *et al.*, 2003; Stover *et al.*, 2000)
ΔphuR	Unmarked deletion of *phuR* in MPAO1 background. Mutant retains the first two and last two codons of the *phuR* gene	This study
ΔphuS	Unmarked deletion of *phuS* in MPAO1 background. Mutant retains the first two and last two codons of the *phuS* gene	This study
ΔphuUV	Unmarked deletion of *phuUV* in MPAO1 background. Mutant retains the first two codons of the *phuU* gene and the last two codons of the *phuV* gene	This study
ΔhasR	Unmarked deletion of *hasR* in MPAO1 background. Mutant retains the first two and last two codons of the *hasR* gene	This study
ΔhxuC	Unmarked deletion of *hxuC* in MPAO1 background. Mutant retains the first two and last two codons of the *hxuC* gene	This study
ΔOMR	Unmarked deletions of *phuR*, *hasR* and *hxuC* in MPAO1 background. Mutant retains the first two and last two codons of each of the deleted genes	This study
ΔcheY	Unmarked deletion of *cheY* in MPAO1 background. Mutant retains the first two and last two codons of the *cheY* gene	This study
ΔprrF1,2	Unmarked deletion of the PrrF1/2 sRNAs in PAO1 background	A.Oglesby-Sherrouse (Wilderman *et al.*, 2004)
ΔPA0028	Unmarked deletion of PA0028 in MPAO1 background. Mutant retains the first three and last three codons of the PA0149 gene	This study
ΔPA0149	Unmarked deletion of PA0149 in MPAO1 background. Mutant retains the first three and last three codons of the PA0149 gene	This study

ΔPA0527	Unmarked deletion of PA0149 in MPAO1 background. Mutant retains the first three and last three codons of the PA0527 gene	This study
ΔPA0709	Unmarked deletion of PA0709 in MPAO1 background. Mutant retains the first three and last three codons of the PA0709 gene	This study
ΔPA3390	Unmarked deletion of PA3390 in MPAO1 background. Mutant retains the first three and last three codons of the PA3390 gene.	This study

**Plasmids**	**Description**	**Source**
pSB109	Medium copy number expression vector for expression of genes under control of the pBAD promoter. Gm^R.	Bardy Lab Ketelboeter *et al*. 2017
pIFP	pSB109 expressing IFP1.4 under the control of the pBAD promoter. Gm^R.	This study
pIFPHO	pSB109 expressing the IFP+HO synthetic operon construct under the control of the pBAD promoter. Gm^R.	This study
pIFPHO_AR	pIFPHO modified to express the *bla* gene from pUCP19. Gm^R and Amp^R.	This study
pBT20	Mariner transposon mutagenesis vector. Gm^R and Amp^R.	Kulasekara *et al*. 2005
pBT20_AS	pBT20 modified to lack the *bla* gene. Gm^R and Amp^S.	This study
pET-15b	Vector for expression of His_6-tagged proteins. Amp^R.	Novagen - EMD Millipore
pET15DG1	pET-15b modified to contain a TEV protease cleavage site in place of the thrombin cleavage site. Amp^R.	This study
pET15DG1 PA0709	pET15DG1 containing PA0709. Amp^R.	This study
pET15DG1 PA3390	pET15DG1 containing PA3390. Amp^R.	This study

**Oligo description**	**Oligo name**	**Nucleotide sequence (5’-3’)**
Primers to poison pSB109 *NcoI* site	*Nco*I poison-F	GTTTCCACGGTGTGCGTCGATGGGCAAATATTAT ACGCAAG
	*Nco*I poison-R	CTTGCGTATAATATTTGCCCATCGACGCACACCGT GGAAAC
Primers for construction of pIFP and pIFPHO	IFP F1	CATGTCACGTGACCCACTTCCATTCTTC
	IFP F2	TCACGTGACCCACTTCCATTCTTC
	IFP R1	TATGTCATTTGTCGTCGTCGTCTTTGTAGTCGAAT TCAGCTTCTTTACGTTGAACTTGAC
	IFP R2	TGTCATTTGTCGTCGTCGTCTTTGTAGTCGAATTC AGCTTCTTTACGTTGAACTTGAC
	IFPHO R1	TATGTCATTTGTCGTCGTCGTCTTTGTAGTCACCT TCTGAAGTAGCAAGACCAACTTC
	IFPHO R2	TGTCATTTGTCGTCGTCGTCTTTGTAGTCACCTTC TGAAGTAGCAAGACCAACTTC

	pSB109 seq F	GAAAAGTCCACATTGATTATTTGCACG
	pSB109 seq R	CGGGCCTCTTCGCTATTACG
Primers for construction of MPAO1 Δ*phuR* mutant	phuR_primer 1	CGATATCCGGCGAGACCACC
	phuR_primer 2	TCAGATGTCGAGCGGCATGTGGGACTC
	phuR_primer 3	ATGCCGCTCGACATCTGACCCGTTACC
	phuR_primer 4	CCAGCTCCACCCAGAGGC
	phuR_primer 5	CCTTCAACGCGGCCCAGC
	phuR_primer 6	GCCGTATCGAAACGCTCC
Primers for construction of MPAO1 Δ*phuS* mutant	phuS_primer 1	CGCTGAAGAAGTCGTTGG
	phuS_primer 2	TCAGAGCGCGCTGCTCATCGACGGTTC
	phuS_primer 3	ATGAGCAGCGCGCTCTGAGGAGAGATC
	phuS_primer 4	CGCGATCCAGTCCGCCTGG
	phuS_primer 5	CGAGGTCAACCAGGAGTGG
	phuS_primer 6	CGGCTTCCGTACTCAGCG
Primers for construction of MPAO1 Δ*phuUV* mutant	phuUV_primer 1	CGTCCTGCTGGTGATCGG
	phuUV_primer 2	TCAACGGGCGACCAACACGAGCGAAAT
	phuUV_primer 3	GTGTTGGTCGCCCGTTGAAGGAGTCGC
	phuUV_primer 4	CGCATGATCTCGCTCCGCT
	phuUV_primer 5	CTGCCGAGGGCGTGCTGG
	phuUV_primer 6	GCATGGCGACTCCTTCAACG
Primers for construction of MPAO1 Δ*hxuC* mutant	hxuC_primer 1	CCGTGGCGGTGAAACAGGG
	hxuC_primer 2	TCAGAAGAACAAGGTCATGCTGGAACT
	hxuC_primer 3	ATGACCTTGTTCTTCTGAGGGGCGTCA
	hxuC_primer 4	GCACCGCCAGTCTCGACC
	hxuC_primer 5	CCTCGCCTACGGCACGGC
	hxuC_primer 6	CCTGCTGCTGCTCAAGAC
Primers for construction of MPAO1 Δ*cheY* mutant	cheY_primer 1	CGACGCGATCCGCGCGATC
	cheY_primer 2	TCAGCCGTTAATTTTCATGTTCTTGTC
	cheY_primer 3	ATGAAAATTAACGGCTGAAGCCGGCCA
	cheY_primer 4	GAGGAACTGTTCGATGCGC
	cheY_primer 5	GGAGCAACTGATCCAGCG

	cheY_primer 6	GCTCCAGCGCGGCCTGGTG
Primers to check PA0028 mutant	PA0028 check F	GACTACCTCTGGTACCGCCT
	PA0028 check R	CGTTCGATGCCAACCAGGAG
Primers to check PA0149 mutant	PA0149 check F	GACTACCTCTGGTACCGCCT
	PA0149 check R	CGTTCGATGCCAACCAGGAG
Primers to check PA0527 mutant	PA0527 check F	GAACTGAACCACATGAGCGGG
	PA0527 check R	CTTCGCGCTGTACGGGTTTTC
Primers to check PA0709 mutant	PA0709 check F	GTGTGGCCGAACACATAGAG
	PA0709 check R	GCGAACCGACCTACTACTCG
Primers to check PA3390 mutant	PA3390 check F	GTTGCCGGATGCTAGGAGG
	PA3390 check R	CATCCGCTCCTGCTTCATGG
Primers to construct PA3390 mutant	PA3390 BamHI F	GGATCCCTTCAGGGAGAGGGGAGAG
	PA3390 Up R	GATTGAGCCTTCTCAGCGCAGGCAGTACACGGAAACTTCCTTTTG
	PA3390 Down F	CAAAAGGAAGTTTCCGTGTA CTGCCTGCGCTGAGAAGGCTCAATC
	PA3390 SpeI R	ACTAGTGACAAGCCGCGACTGAAGG
Primers for construction of pUCP19-phuUV complement plasmid	phuUV_Forward	GATACAAGCTTCGAAACCGCTGAGTACG
	phuUV_Reverse	GCTAGGGATCCTCAACGGGCGACGATCAG
Primers for amplification of pUCP19 *bla*	pUCP19_bla_F	CTAGACTGCAGCGTCAGGTGGCACTTTTCG
	pUCP19_bla_R	CGATGCTGCAGGGATCTCAAGAAGATCCTTTGATC
Primers used in *Tn*-seq analysis	pBT20-92_1	GAGCATCGTTTGTTCGCCCGGCTTCTGTATGG
	olj376	GTGACTGGAGTTCAGACGTGTGCTCTTCCGATCTGGGGGGGGGGGGGGGG

	pBT20_PAIR_AmpF_2	AATGATACGGCGACCACCGAGATCTACACTCTTTCGTCTAGAAGCCTGCTTTCTAGAGACCGGGGACTTATCAGC
	TdT_Index_1_ATCACG	CAAGCAGAAGACGGCATACGAGATCGTGATGTGACTGGAGTTCAGACGTGTGCTCTTCCGATCT
	TdT_Index_3_TTAGGC	CAAGCAGAAGACGGCATACGAGATGCCTAAGTGACTGGAGTTCAGACGTGTGCTCTTCCGATCT
	TdT_Index_4_TGACCA	CAAGCAGAAGACGGCATACGAGATTGGTCAGTGACTGGAGTTCAGACGTGTGCTCTTCCGATCT
	pBT20_SEQ-6	CCTGCTTTCTAGAGACCGGGGACTTATCAGCCAAC
Primers for construction of pET15DG1	pET15b TEV F	CATGGGCAGCAGCCATCATCATCATCATCACAGCAGCGGCGAGAATCTTTATTTTCAGGGACA
	pET15b TEV R	TATGTCCCTGAAAATAAAGATTCTCGCCGCTGCTGTGATGATGATGATGATGGCTGCTGCC
Primers for construction of PA0709 and PA3390 expression plasmids	PA0709-F1	TATGACCTACCACGTACTGGTTC
	PA0709-F2	TGACCTACCACGTACTGGTTC
	PA0709-R1	GATCCTCAACCCTCGACGCGGTAG
	PA0709-R2	CTCAACCCTCGACGCGGTAG
	PA3390-F1	TATGTACTGCATCTTTATCAAGGCC
	PA3390-F2	TGTACTGCATCTTTATCAAGGCC
	PA3390-R1	GATCCTCAGCGCAGCGGATTGAGC
	PA3390-R2	CTCAGCGCAGCGGATTGAGC
